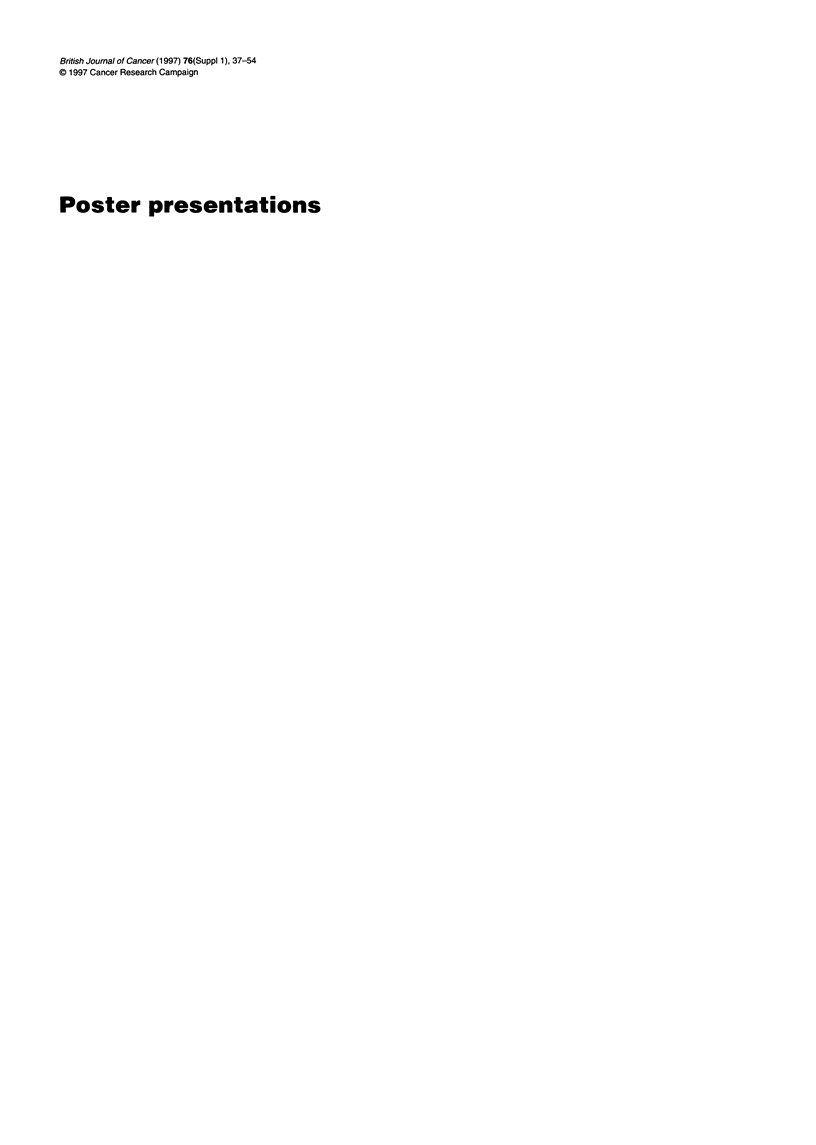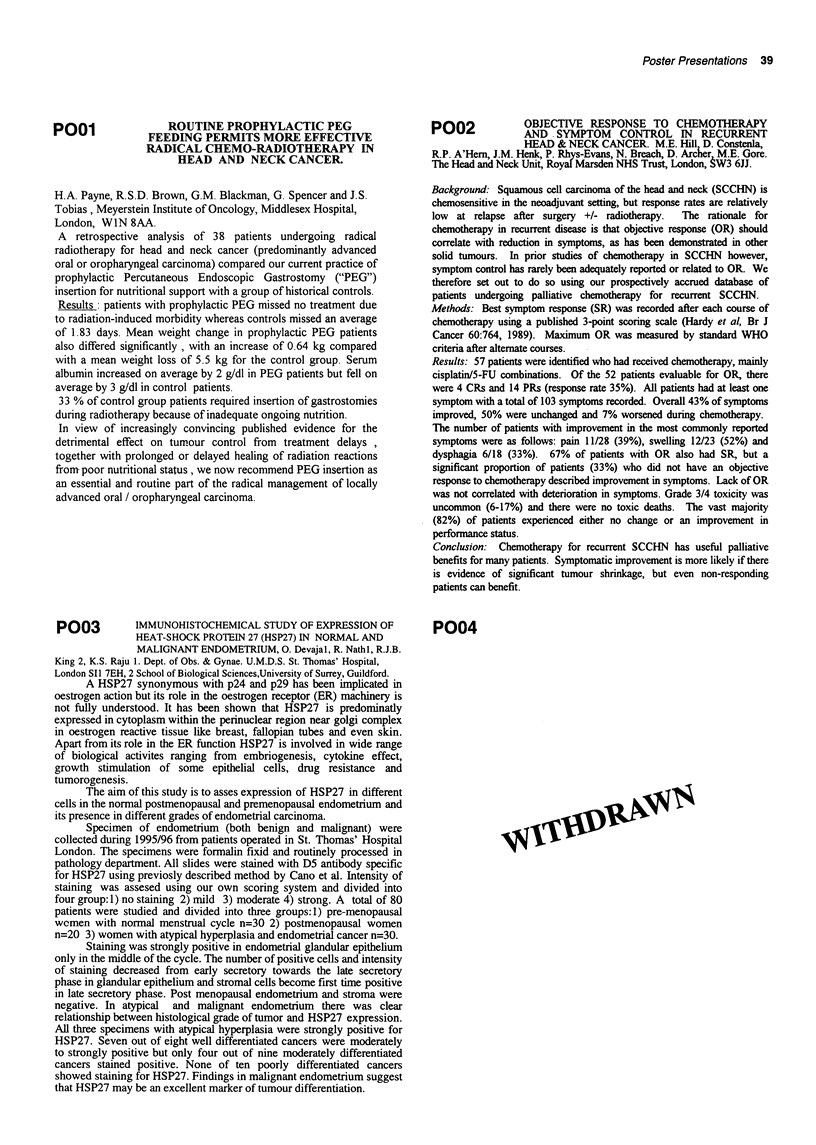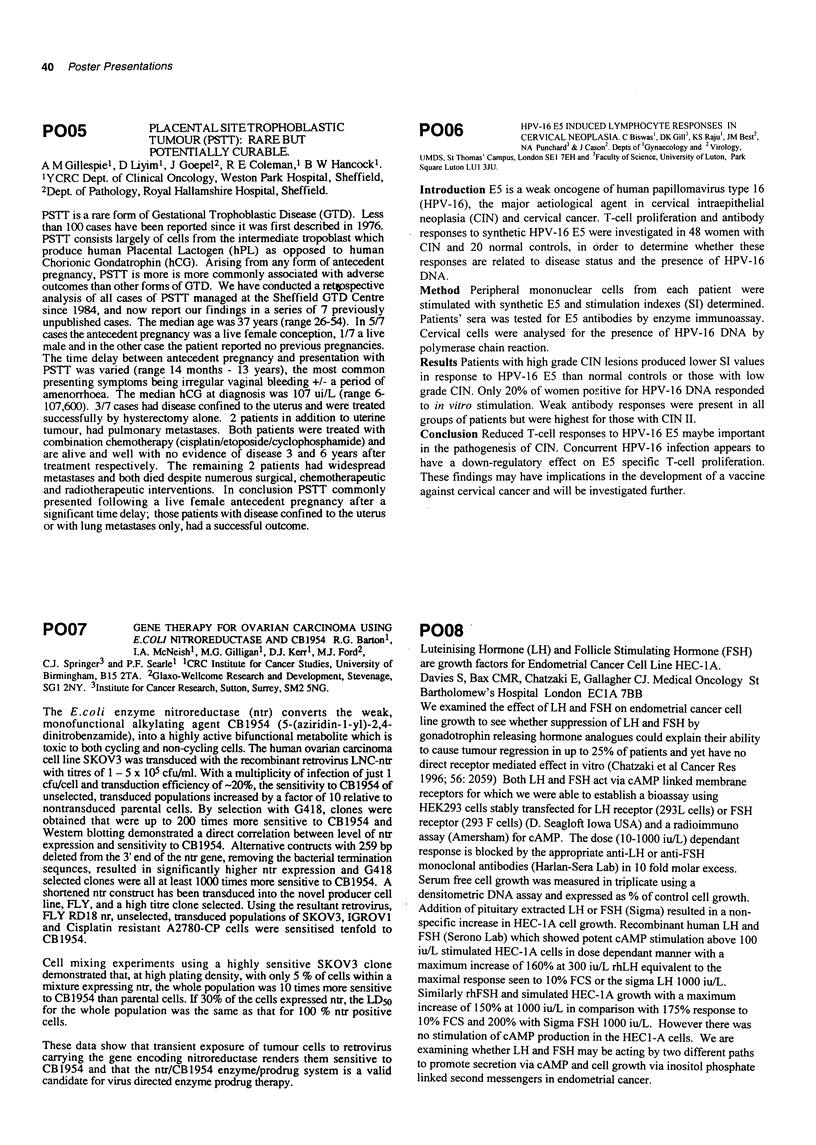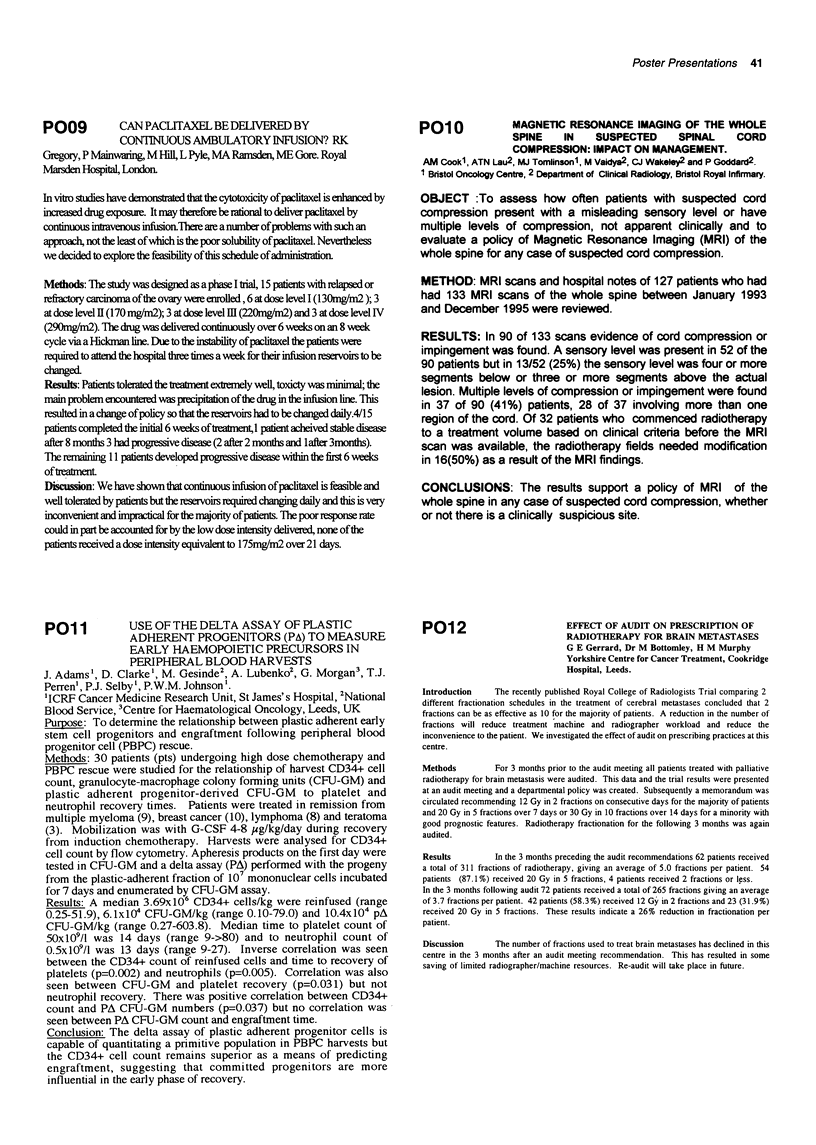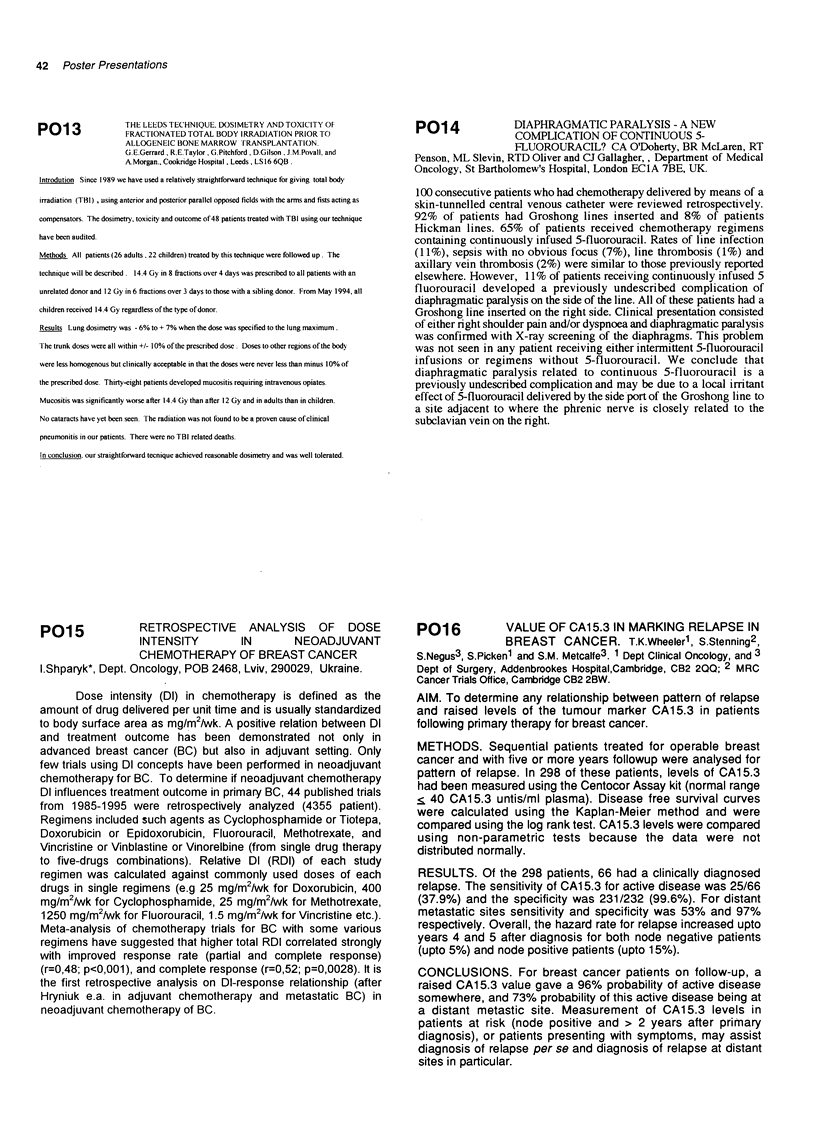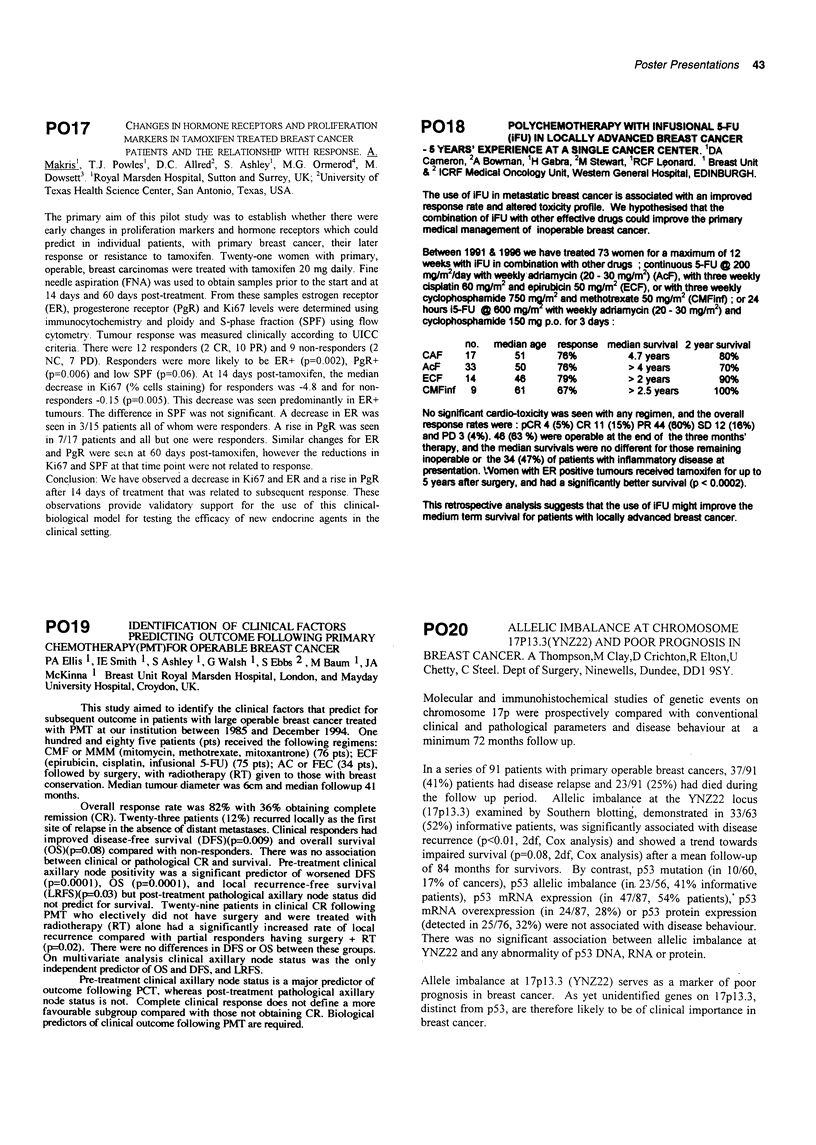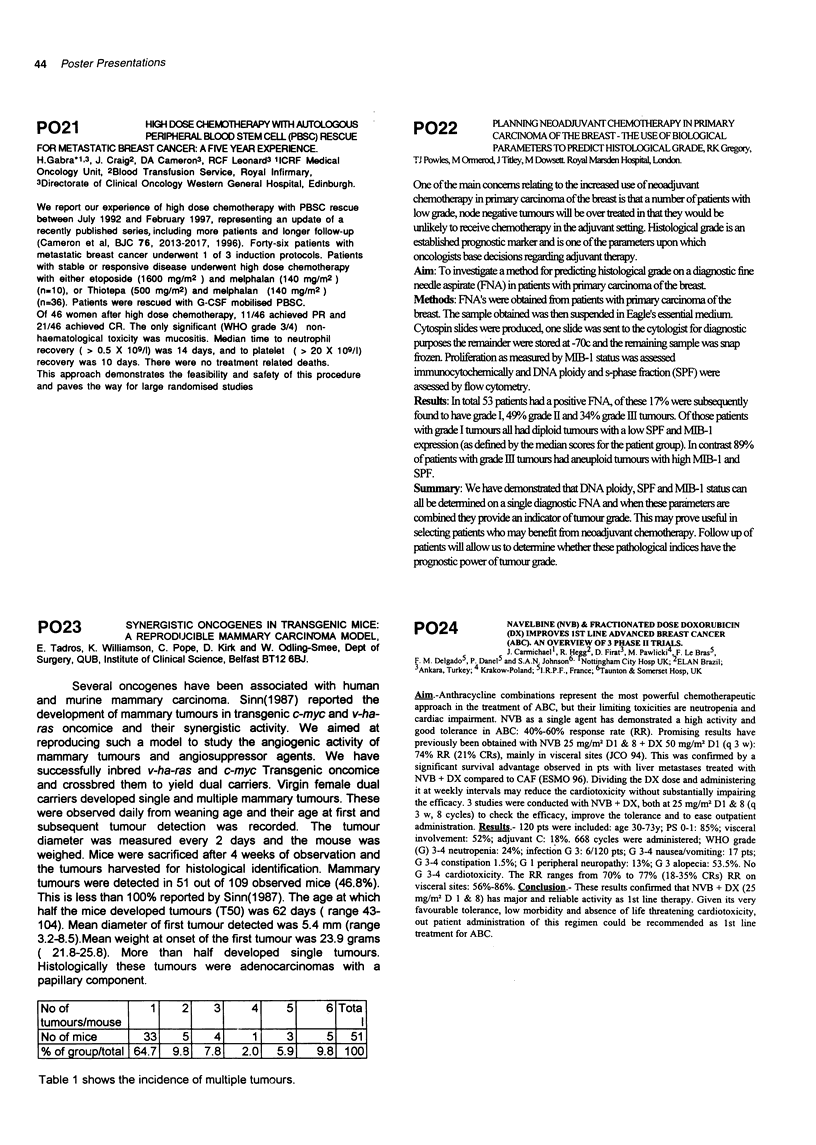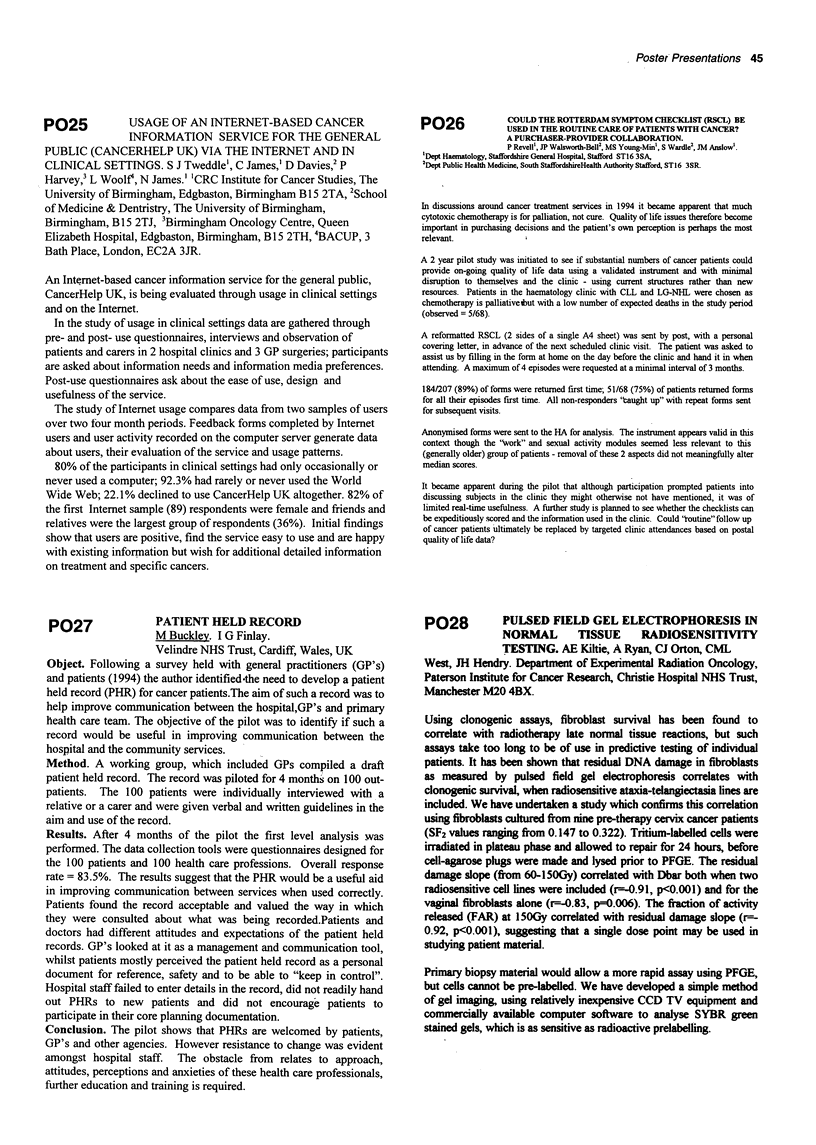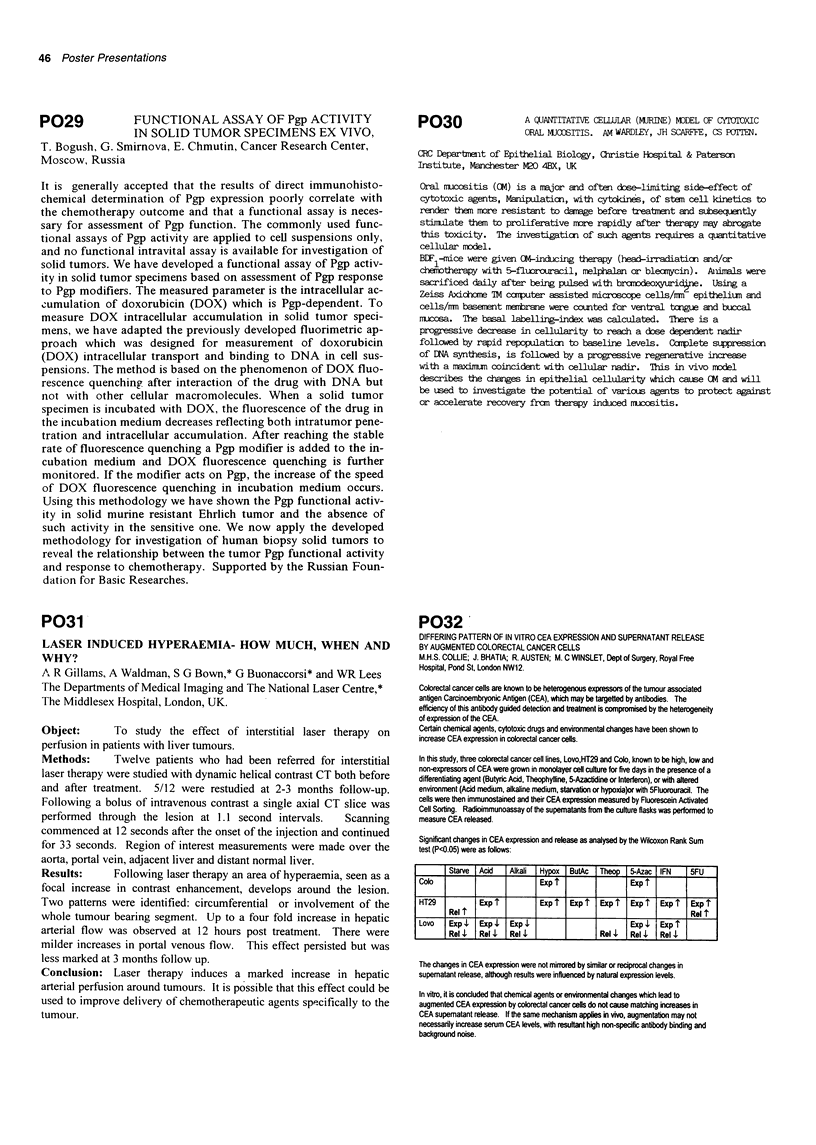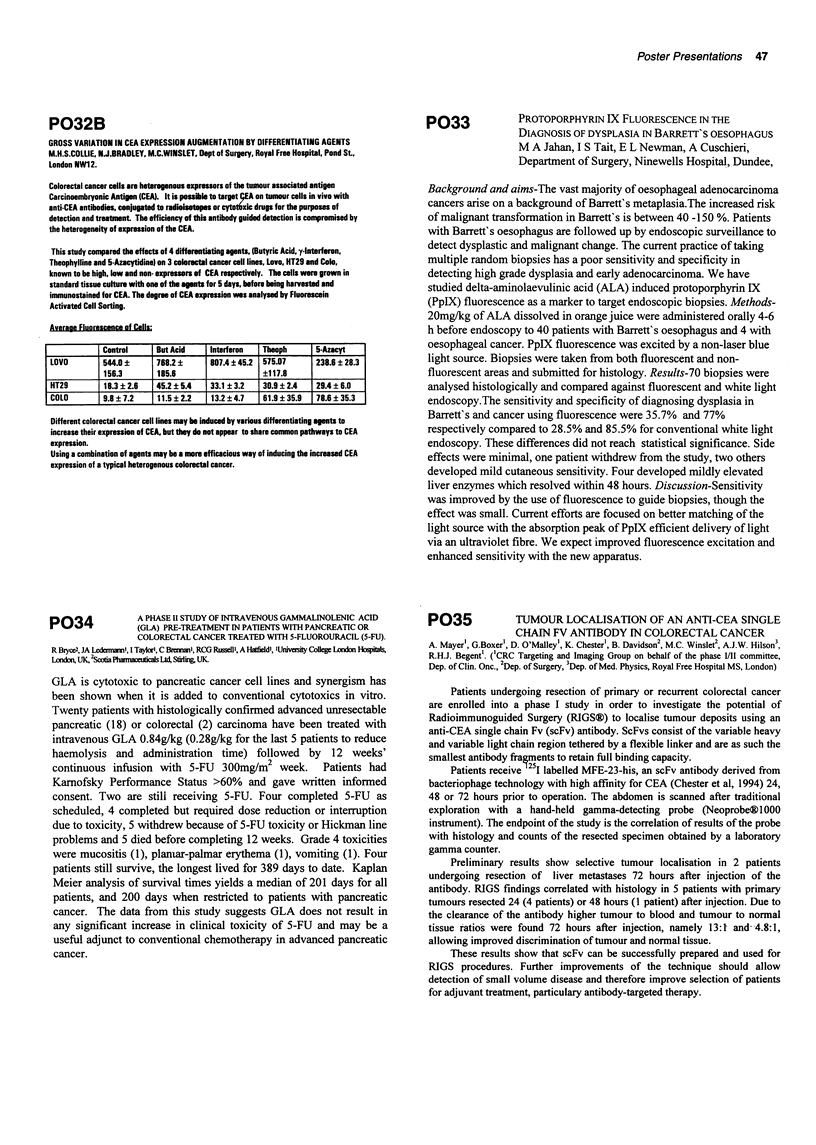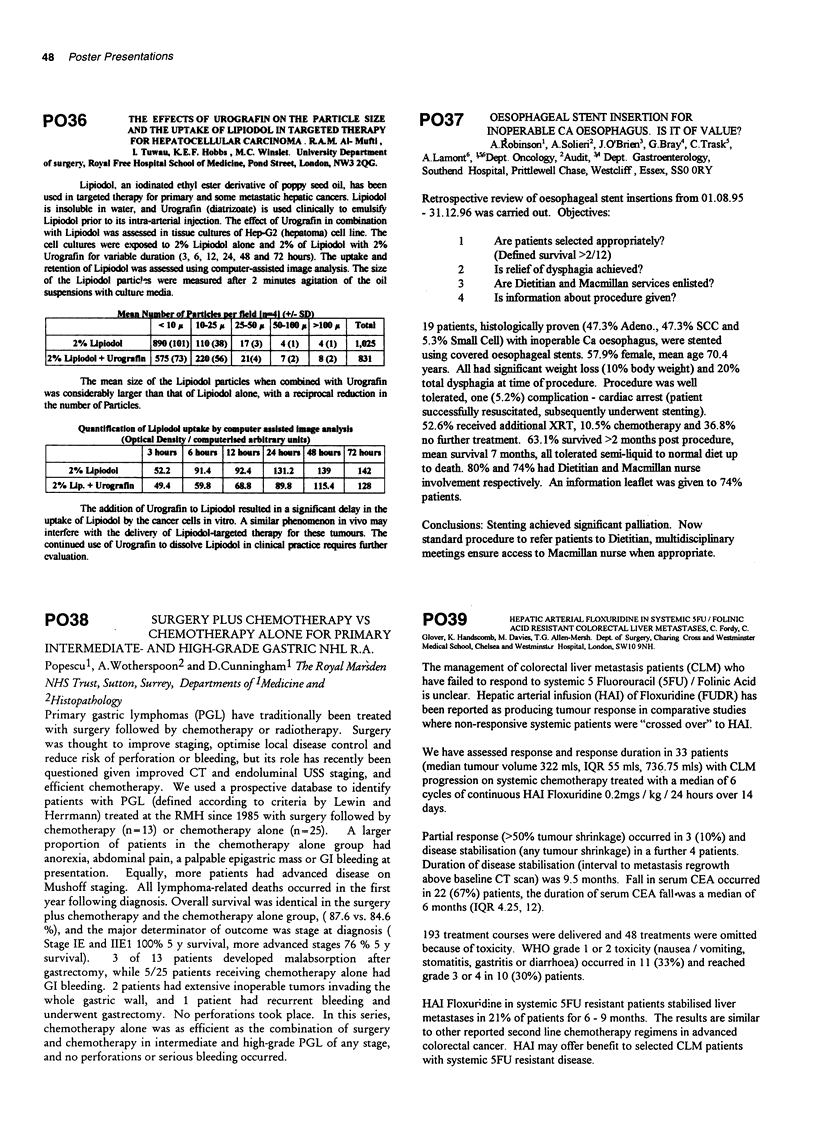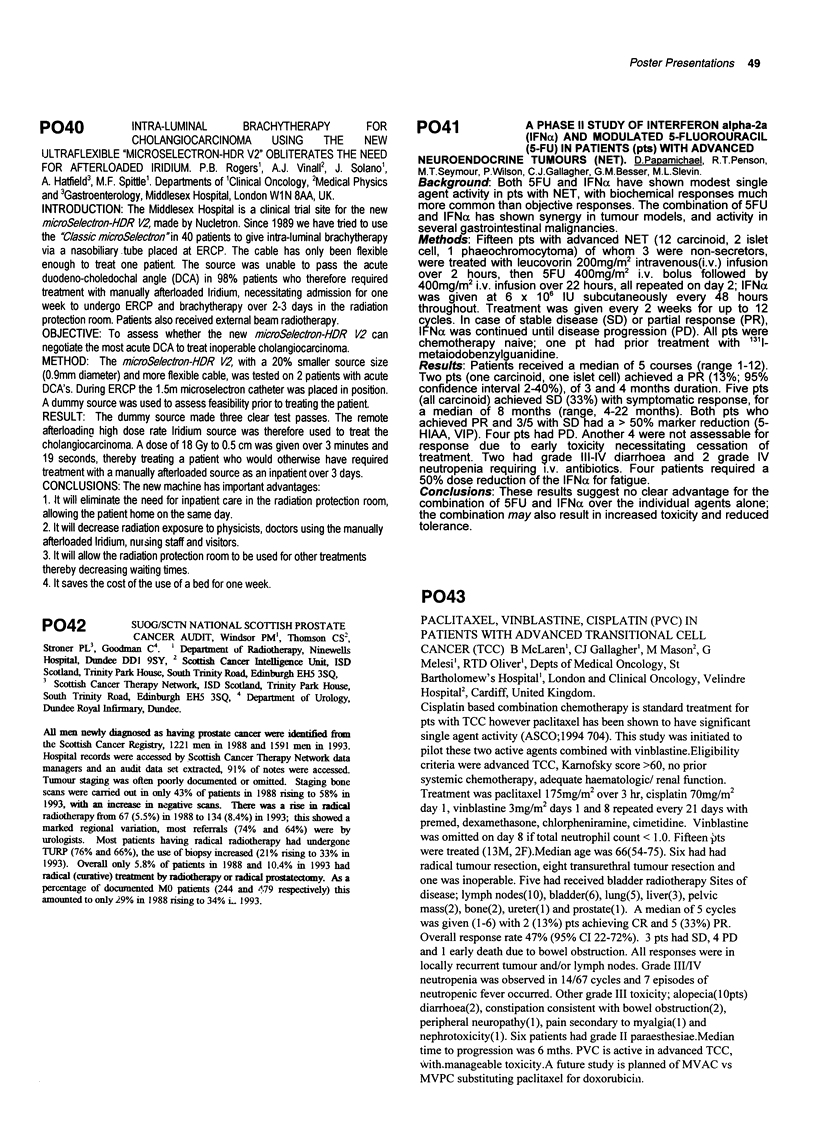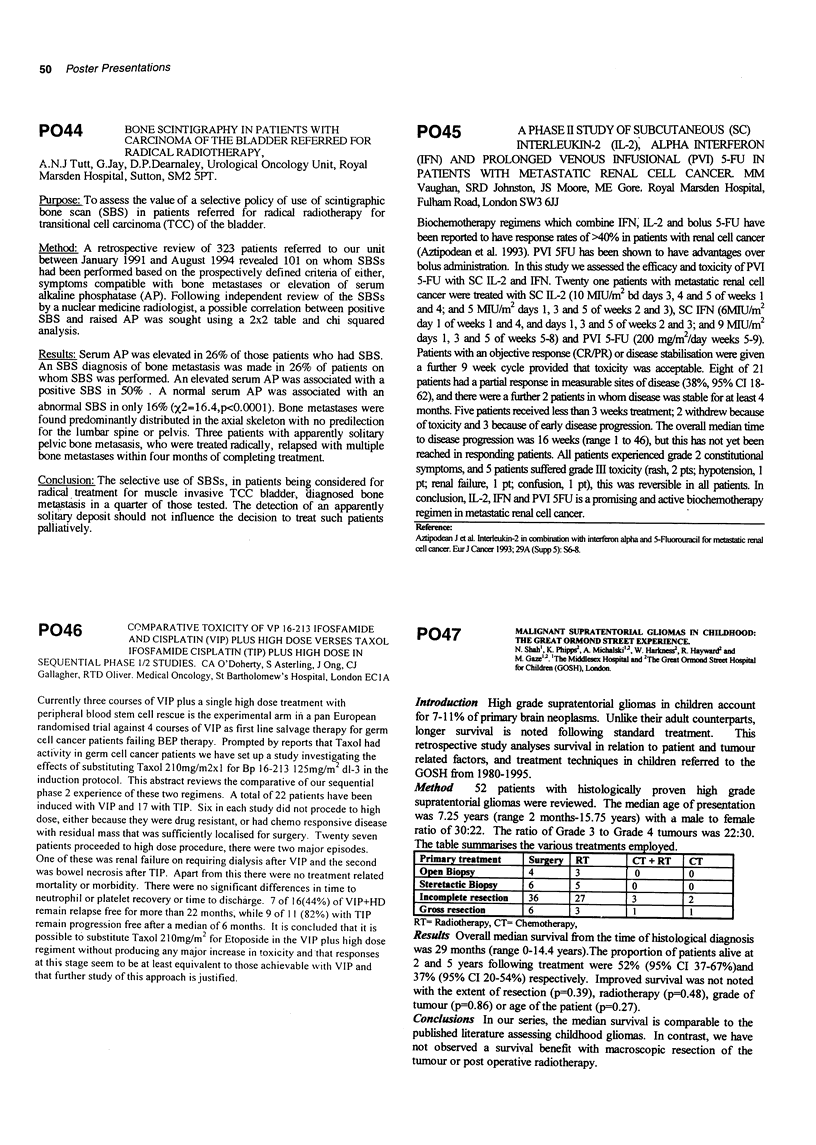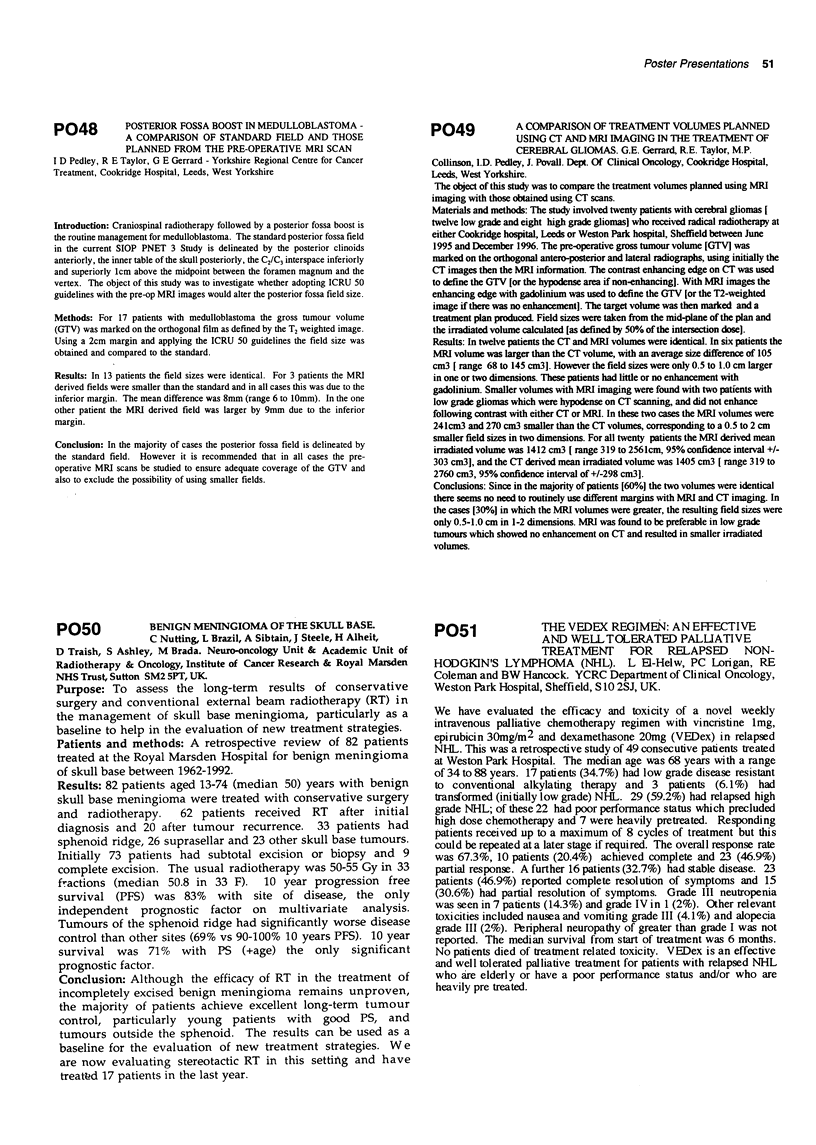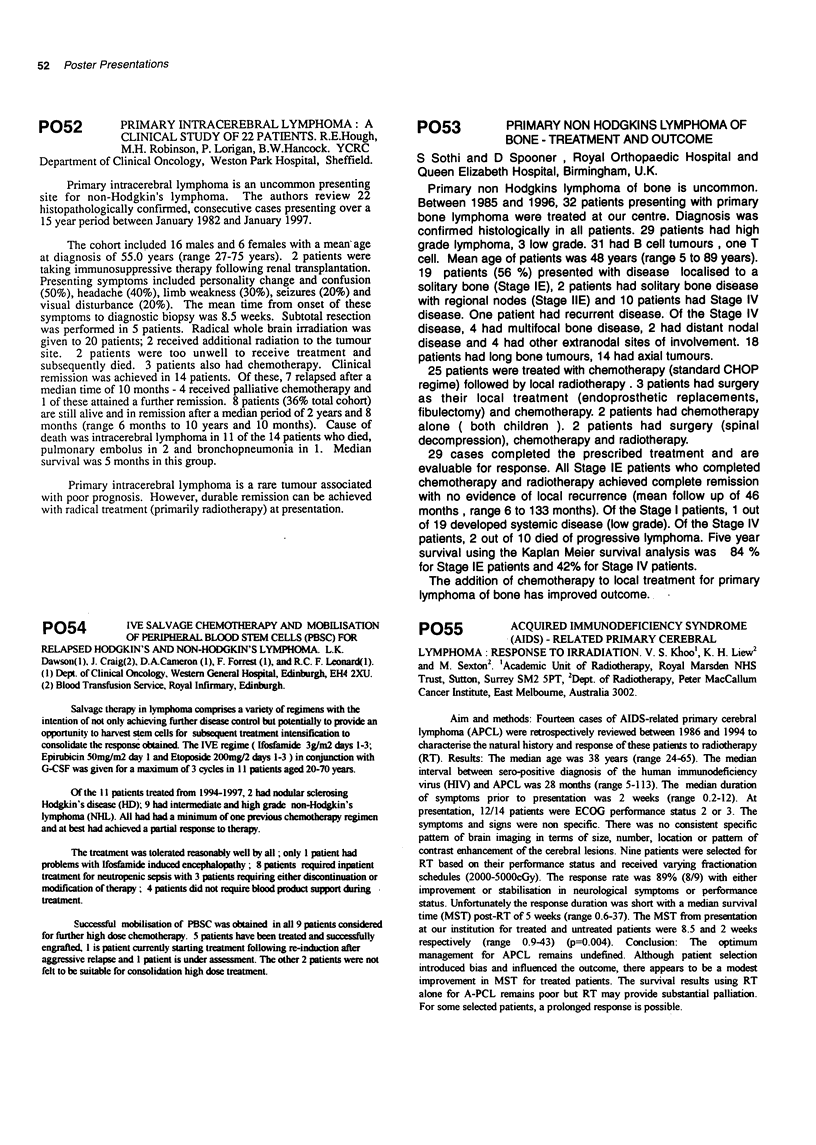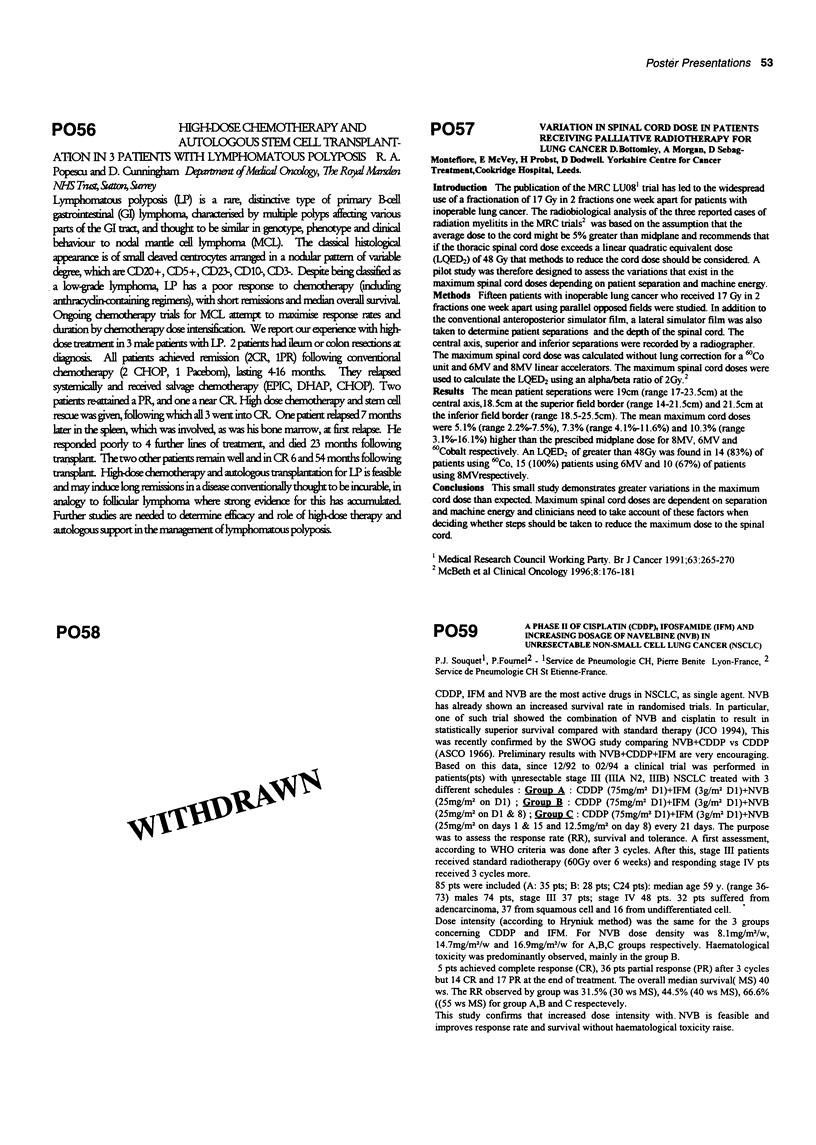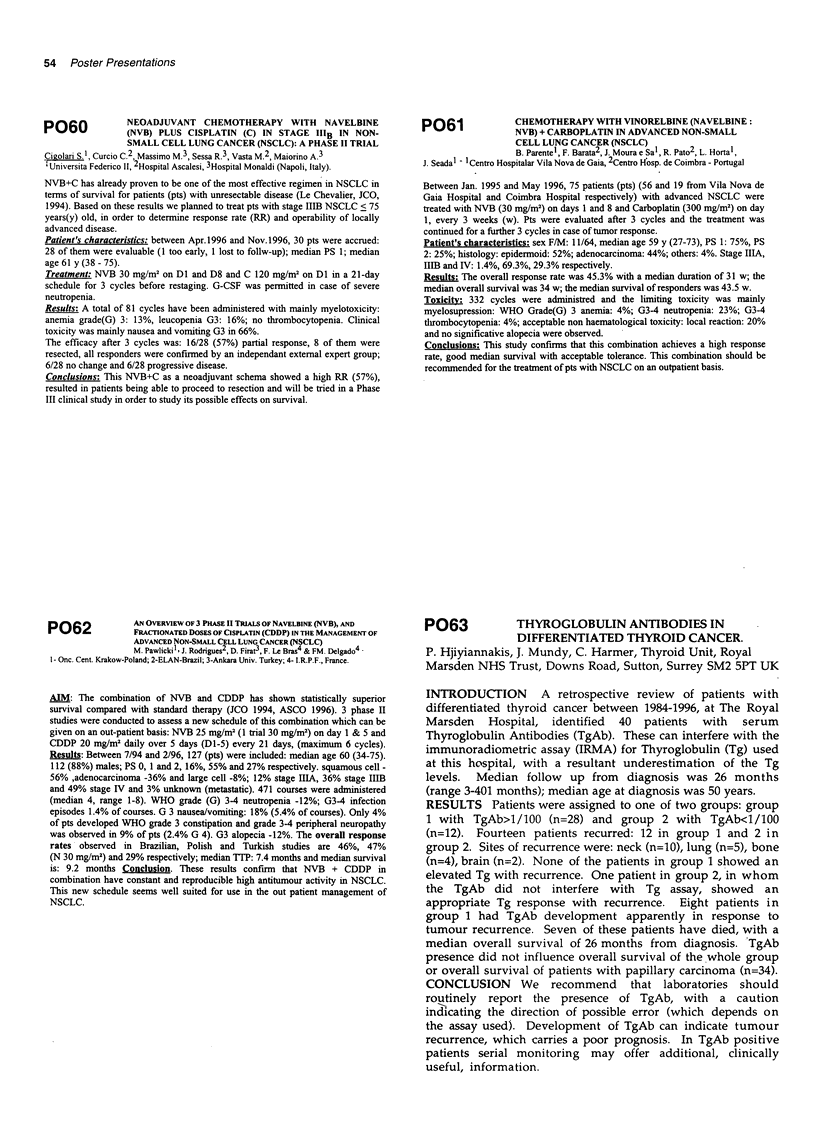# Poster presentations

**Published:** 1997

**Authors:** 


*British Journal of Cancer* (1997) **76**(Suppl 1), 37–54
© 1997 Cancer Research Campaign

### Poster presentations

### PO01 ROUTINE PROPHYLACTIC PEG FEEDING PERMITS MORE EFFECTIVE RADICAL CHEMO-RADIOTHERAPY IN HEAD AND NECK CANCER.

H.A. Payne, R.S.D. Brown, G.M. Blackman, G. Spencer and J.S. Tobias , Meyerstein Institute of Oncology, Middlesex Hospital, London, W1N 8AA.

A retrospective analysis of 38 patients undergoing radical radiotherapy for head and neck cancer (predominantly advanced oral or oropharyngeal carcinoma) compared our current practice of prophylactic Percutaneous Endoscopic Gastrostomy ("PEG") insertion for nutritional support with a group of historical controls.

Results : patients with prophylactic PEG missed no treatment due to radiation-induced morbidity whereas controls missed an average of 1.83 days. Mean weight change in prophylactic PEG patients also differed significantly , with an increase of 0.64 kg compared with a mean weight loss of 5.5 kg for the control group. Serum albumin increased on average by 2 g/dl in PEG patients but fell on average by 3 g/dl in control patients.

33 % of control group patients required insertion of gastrostomies during radiotherapy because of inadequate ongoing nutrition.

In view of increasingly convincing published evidence for the detrimental effect on tumour control from treatment delays , together with prolonged or delayed healing of radiation reactions from poor nutritional status , we now recommend PEG insertion as an essential and routine part of the radical management of locally advanced oral / oropharyngeal carcinoma.

### PO02 OBJECTIVE RESPONSE TO CHEMOTHERAPY AND SYMPTOM CONTROL IN RECURRENT HEAD & NECK CANCER.

M.E. Hill, D. Constenla, R.P. A'Hern, J.M. Henk, P. Rhys-Evans, N. Breach, D. Archer, M.E. Gore. The Head and Neck Unit, Royal Marsden NHS Trust, London, SW3 6JJ.

*Background:* Squamous cell carcinoma of the head and neck (SCCHN) is chemosensitive in the neoadjuvant setting, but response rates are relatively low at relapse after surgery +/- radiotherapy. The rationale for chemotherapy in recurrent disease is that objective response (OR) should correlate with reduction in symptoms, as has been demonstrated in other solid tumours. In prior studies of chemotherapy in SCCHN however, symptom control has rarely been adequately reported or related to OR. We therefore set out to do so using our prospectively accrued database of patients undergoing palliative chemotherapy for recurrent SCCHN.

*Methods:* Best symptom response (SR) was recorded after each course of chemotherapy using a published 3-point scoring scale (Hardy *et al,* Br J Cancer 60:764, 1989). Maximum OR was measured by standard WHO criteria after alternate courses.

*Results:* 57 patients were identified who had received chemotherapy, mainly cisplatin/5-FU combinations. Of the 52 patients evaluable for OR, there were 4 CRs and 14 PRs (response rate 35%). All patients had at least one symptom with a total of 103 symptoms recorded. Overall 43% of symptoms improved, 50% were unchanged and 7% worsened during chemotherapy. The number of patients with improvement in the most commonly reported symptoms were as follows: pain 11/28 (39%), swelling 12/23 (52%) and dysphagia 6/18 (33%). 67% of patients with OR also had SR, but a significant proportion of patients (33%) who did not have an objective response to chemotherapy described improvement in symptoms. Lack of OR was not correlated with deterioration in symptoms. Grade 3/4 toxicity was uncommon (6-17%) and there were no toxic deaths. The vast majority (82%) of patients experienced either no change or an improvement in performance status.

*Conclusion:* Chemotherapy for recurrent SCCHN has useful palliative benefits for many patients. Symptomatic improvement is more likely if there is evidence of significant tumour shrinkage, but even non-responding patients can benefit.

### PO03 IMMUNOHISTOCHEMICAL STUDY OF EXPRESSION OF HEAT-SHOCK PROTEIN 27 (HSP27) IN NORMAL AND MALIGNANT ENDOMETRIUM,

O. Devaja1, R. Nath1, R.J.B. King 2, K.S. Raju 1. Dept. of Obs. & Gynae. U.M.D.S. St. Thomas' Hospital, London SI1 7EH, 2 School of Biological Sciences,University of Surrey, Guildford.

A HSP27 synonymous with p24 and p29 has been implicated in oestrogen action but its role in the oestrogen receptor (ER) machinery is not fully understood. It has been shown that HSP27 is predominatly expressed in cytoplasm within the perinuclear region near golgi complex in oestrogen reactive tissue like breast, fallopian tubes and even skin. Apart from its role in the ER function HSP27 is involved in wide range of biological activites ranging from embriogenesis, cytokine effect, growth stimulation of some epithelial cells, drug resistance and tumorogenesis.

The aim of this study is to asses expression of HSP27 in different cells in the normal postmenopausal and premenopausal endometrium and its presence in different grades of endometrial carcinoma.

Specimen of endometrium (both benign and malignant) were collected during 1995/96 from patients operated in St. Thomas' Hospital London. The specimens were formalin fixid and routinely processed in pathology department. All slides were stained with D5 antibody specific for HSP27 using previously described method by Cano et al. Intensity of staining was assesed using our own scoring system and divided into four group:1) no staining 2) mild 3) moderate 4) strong. A total of 80 patients were studied and divided into three groups:1) pre-menopausal women with normal menstrual cycle n=30 2) postmenopausal women n=20 3) women with atypical hyperplasia and endometrial cancer n=30.

Staining was strongly positive in endometrial glandular epithelium only in the middle of the cycle. The number of positive cells and intensity of staining decreased from early secretory towards the late secretory phase in glandular epithelium and stromal cells become first time positive in late secretory phase. Post menopausal endometrium and stroma were negative. In atypical and malignant endometrium there was clear relationship between histological grade of tumor and HSP27 expression. All three specimens with atypical hyperplasia were strongly positive for HSP27. Seven out of eight well differentiated cancers were moderately to strongly positive but only four out of nine moderately differentiated cancers stained positive. None of ten poorly differentiated cancers showed staining for HSP27. Findings in malignant endometrium suggest that HSP27 may be an excellent marker of tumour differentiation.

### PO04

WITHDRAWN

### PO05

PLACENTAL SITE TROPHOBLASTIC TUMOUR (PSTT): RARE BUT POTENTIALLY CURABLE.

A M Gillespie[1], D Liyim[1], J Goepel[2], R E Coleman,[1] B W Hancock[1].

[1]YCRC Dept. of Clinical Oncology, Weston Park Hospital, Sheffield,

[2]Dept. of Pathology, Royal Hallamshire Hospital, Sheffield.

PSTT is a rare form of Gestational Trophoblastic Disease (GTD). Less than 100 cases have been reported since it was first described in 1976. PSTT consists largely of cells from the intermediate tropoblast which produce human Placental Lactogen (hPL) as opposed to human Chorionic Gondatrophin (hCG). Arising from any form of antecedent pregnancy, PSTT is more is more commonly associated with adverse outcomes than other forms of GTD. We have conducted a retrospective analysis of all cases of PSTT managed at the Sheffield GTD Centre since 1984, and now report our findings in a series of 7 previously unpublished cases. The median age was 37 years (range 26-54). In 5/7 cases the antecedent pregnancy was a live female conception, 1/7 a live male and in the other case the patient reported no previous pregnancies. The time delay between antecedent pregnancy and presentation with PSTT was varied (range 14 months - 13 years), the most common presenting symptoms being irregular vaginal bleeding +/- a period of amenorrhoea. The median hCG at diagnosis was 107 ui/L (range 6-107,600). 3/7 cases had disease confined to the uterus and were treated successfully by hysterectomy alone. 2 patients in addition to uterine tumour, had pulmonary metastases. Both patients were treated with combination chemotherapy (cisplatin/etoposide/cyclophosphamide) and are alive and well with no evidence of disease 3 and 6 years after treatment respectively. The remaining 2 patients had widespread metastases and both died despite numerous surgical, chemotherapeutic and radiotherapeutic interventions. In conclusion PSTT commonly presented following a live female antecedent pregnancy after a significant time delay; those patients with disease confined to the uterus or with lung metastases only, had a successful outcome.

### PO06

HPV-16 E5 INDUCED LYMPHOCYTE RESPONSES IN CERVICAL NEOPLASIA.

C Biswas[1], DK Gill[3], KS Raju[1], JM Best[2], NA Punchard[3] & J Cason[2]. Depts of [1]Gynaecology and [2]Virology, UMDS, St Thomas' Campus, London SE1 7EH and [3]Faculty of Science, University of Luton, Park Square Luton LU1 3JU.

**Introduction** E5 is a weak oncogene of human papillomavirus type 16 (HPV-16), the major aetiological agent in cervical intraepithelial neoplasia (CIN) and cervical cancer. T-cell proliferation and antibody responses to synthetic HPV-16 E5 were investigated in 48 women with CIN and 20 normal controls, in order to determine whether these responses are related to disease status and the presence of HPV-16 DNA.

**Method** Peripheral mononuclear cells from each patient were stimulated with synthetic E5 and stimulation indexes (SI) determined. Patients' sera was tested for E5 antibodies by enzyme immunoassay. Cervical cells were analysed for the presence of HPV-16 DNA by polymerase chain reaction.

**Results** Patients with high grade CIN lesions produced lower SI values in response to HPV-16 E5 than normal controls or those with low grade CIN. Only 20% of women positive for HPV-16 DNA responded to *in vitro* stimulation. Weak antibody responses were present in all groups of patients but were highest for those with CIN II.

**Conclusion** Reduced T-cell responses to HPV-16 E5 maybe important in the pathogenesis of CIN. Concurrent HPV-16 infection appears to have a down-regulatory effect on E5 specific T-cell proliferation. These findings may have implications in the development of a vaccine against cervical cancer and will be investigated further.

### PO07

GENE THERAPY FOR OVARIAN CARCINOMA USING *E.COLI* NITROREDUCTASE AND CB1954

R.G. Barton[1], I.A. McNeish[1], M.G. Gilligan[1], D.J. Kerr[1], M.J. Ford[2], C.J. Springer[3] and P.F. Searle[1] [1]CRC Institute for Cancer Studies, University of Birmingham, B15 2TA. [2]Glaxo-Wellcome Research and Development, Stevenage, SG1 2NY. [3]Institute for Cancer Research, Sutton, Surrey, SM2 5NG.

The *E.coli* enzyme nitroreductase (ntr) converts the weak, monofunctional alkylating agent CB1954 (5-(aziridin-1-yl)-2,4-dinitrobenzamide), into a highly active bifunctional metabolite which is toxic to both cycling and non-cycling cells. The human ovarian carcinoma cell line SKOV3 was transduced with the recombinant retrovirus LNC-ntr with titres of 1 - 5 x $10^5$ cfu/ml. With a multiplicity of infection of just 1 cfu/cell and transduction efficiency of ~20%, the sensitivity to CB1954 of unselected, transduced populations increased by a factor of 10 relative to nontransduced parental cells. By selection with G418, clones were obtained that were up to 200 times more sensitive to CB1954 and Western blotting demonstrated a direct correlation between level of ntr expression and sensitivity to CB1954. Alternative contructs with 259 bp deleted from the 3' end of the ntr gene, removing the bacterial termination sequnces, resulted in significantly higher ntr expression and G418 selected clones were all at least 1000 times more sensitive to CB1954. A shortened ntr construct has been transduced into the novel producer cell line, FLY, and a high titre clone selected. Using the resultant retrovirus, FLY RD18 nr, unselected, transduced populations of SKOV3, IGROV1 and Cisplatin resistant A2780-CP cells were sensitised tenfold to CB1954.

Cell mixing experiments using a highly sensitive SKOV3 clone demonstrated that, at high plating density, with only 5 % of cells within a mixture expressing ntr, the whole population was 10 times more sensitive to CB1954 than parental cells. If 30% of the cells expressed ntr, the $LD_{50}$ for the whole population was the same as that for 100 % ntr positive cells.

These data show that transient exposure of tumour cells to retrovirus carrying the gene encoding nitroreductase renders them sensitive to CB1954 and that the ntr/CB1954 enzyme/prodrug system is a valid candidate for virus directed enzyme prodrug therapy.

### PO08

Luteinising Hormone (LH) and Follicle Stimulating Hormone (FSH) are growth factors for Endometrial Cancer Cell Line HEC-1A.

Davies S, Bax CMR, Chatzaki E, Gallagher CJ. Medical Oncology St Bartholomew's Hospital London EC1A 7BB

We examined the effect of LH and FSH on endometrial cancer cell line growth to see whether suppression of LH and FSH by gonadotrophin releasing hormone analogues could explain their ability to cause tumour regression in up to 25% of patients and yet have no direct receptor mediated effect in vitro (Chatzaki et al Cancer Res 1996; 56: 2059) Both LH and FSH act via cAMP linked membrane receptors for which we were able to establish a bioassay using HEK293 cells stably transfected for LH receptor (293L cells) or FSH receptor (293 F cells) (D. Seagloft Iowa USA) and a radioimmuno assay (Amersham) for cAMP. The dose (10-1000 iu/L) dependant response is blocked by the appropriate anti-LH or anti-FSH monoclonal antibodies (Harlan-Sera Lab) in 10 fold molar excess. Serum free cell growth was measured in triplicate using a densitometric DNA assay and expressed as % of control cell growth. Addition of pituitary extracted LH or FSH (Sigma) resulted in a non-specific increase in HEC-1A cell growth. Recombinant human LH and FSH (Serono Lab) which showed potent cAMP stimulation above 100 iu/L stimulated HEC-1A cells in dose dependant manner with a maximum increase of 160% at 300 iu/L rhLH equivalent to the maximal response seen to 10% FCS or the sigma LH 1000 iu/L. Similarly rhFSH and simulated HEC-1A growth with a maximum increase of 150% at 1000 iu/L in comparison with 175% response to 10% FCS and 200% with Sigma FSH 1000 iu/L. However there was no stimulation of cAMP production in the HEC1-A cells. We are examining whether LH and FSH may be acting by two different paths to promote secretion via cAMP and cell growth via inositol phosphate linked second messengers in endometrial cancer.

### PO09 CAN PACLITAXEL BE DELIVERED BY CONTINUOUS AMBULATORY INFUSION?

RK Gregory, P Mainwaring, M Hill, L Pyle, MA Ramsden, ME Gore. Royal Marsden Hospital, London.

In vitro studies have demonstrated that the cytotoxicity of paclitaxel is enhanced by increased drug exposure. It may therefore be rational to deliver paclitaxel by continuous intravenous infusion.There are a number of problems with such an approach, not the least of which is the poor solubility of paclitaxel. Nevertheless we decided to explore the feasibility of this schedule of administration.

**Methods**: The study was designed as a phase I trial, 15 patients with relapsed or refractory carcinoma of the ovary were enrolled , 6 at dose level I (130mg/m2 ); 3 at dose level II (170 mg/m2); 3 at dose level III (220mg/m2) and 3 at dose level IV (290mg/m2). The drug was delivered continuously over 6 weeks on an 8 week cycle via a Hickman line. Due to the instability of paclitaxel the patients were required to attend the hospital three times a week for their infusion reservoirs to be changed.

**Results**: Patients tolerated the treatment extremely well, toxicty was minimal; the main problem encountered was precipitation of the drug in the infusion line. This resulted in a change of policy so that the reservoirs had to be changed daily.4/15 patients completed the initial 6 weeks of treatment,1 patient acheived stable disease after 8 months 3 had progressive disease (2 after 2 months and 1after 3months). The remaining 11 patients developed progressive disease within the first 6 weeks of treatment.

**Discussion**: We have shown that continuous infusion of paclitaxel is feasible and well tolerated by patients but the reservoirs required changing daily and this is very inconvenient and impractical for the majority of patients. The poor response rate could in part be accounted for by the low dose intensity delivered, none of the patients received a dose intensity equivalent to 175mg/m2 over 21 days.

### PO10 MAGNETIC RESONANCE IMAGING OF THE WHOLE SPINE IN SUSPECTED SPINAL CORD COMPRESSION: IMPACT ON MANAGEMENT.

AM Cook[1], ATN Lau[2], MJ Tomlinson[1], M Vaidya[2], CJ Wakeley[2] and P Goddard[2].
[1] Bristol Oncology Centre, [2] Department of Clinical Radiology, Bristol Royal Infirmary.

**OBJECT** :To assess how often patients with suspected cord compression present with a misleading sensory level or have multiple levels of compression, not apparent clinically and to evaluate a policy of Magnetic Resonance Imaging (MRI) of the whole spine for any case of suspected cord compression.

**METHOD**: MRI scans and hospital notes of 127 patients who had had 133 MRI scans of the whole spine between January 1993 and December 1995 were reviewed.

**RESULTS:** In 90 of 133 scans evidence of cord compression or impingement was found. A sensory level was present in 52 of the 90 patients but in 13/52 (25%) the sensory level was four or more segments below or three or more segments above the actual lesion. Multiple levels of compression or impingement were found in 37 of 90 (41%) patients, 28 of 37 involving more than one region of the cord. Of 32 patients who commenced radiotherapy to a treatment volume based on clinical criteria before the MRI scan was available, the radiotherapy fields needed modification in 16(50%) as a result of the MRI findings.

**CONCLUSIONS**: The results support a policy of MRI of the whole spine in any case of suspected cord compression, whether or not there is a clinically suspicious site.

### PO11 USE OF THE DELTA ASSAY OF PLASTIC ADHERENT PROGENITORS (PΔ) TO MEASURE EARLY HAEMOPOIETIC PRECURSORS IN PERIPHERAL BLOOD HARVESTS

J. Adams[1], D. Clarke[1], M. Gesinde[2], A. Lubenko[2], G. Morgan[3], T.J. Perren[1], P.J. Selby[1], P.W.M. Johnson[1].

[1]ICRF Cancer Medicine Research Unit, St James's Hospital, [2]National Blood Service, [3]Centre for Haematological Oncology, Leeds, UK

Purpose: To determine the relationship between plastic adherent early stem cell progenitors and engraftment following peripheral blood progenitor cell (PBPC) rescue.

Methods: 30 patients (pts) undergoing high dose chemotherapy and PBPC rescue were studied for the relationship of harvest CD34+ cell count, granulocyte-macrophage colony forming units (CFU-GM) and plastic adherent progenitor-derived CFU-GM to platelet and neutrophil recovery times. Patients were treated in remission from multiple myeloma (9), breast cancer (10), lymphoma (8) and teratoma (3). Mobilization was with G-CSF 4-8 $\mu$g/kg/day during recovery from induction chemotherapy. Harvests were analysed for CD34+ cell count by flow cytometry. Apheresis products on the first day were tested in CFU-GM and a delta assay (PΔ) performed with the progeny from the plastic-adherent fraction of $10^7$ mononuclear cells incubated for 7 days and enumerated by CFU-GM assay.

Results: A median $3.69\times10^6$ CD34+ cells/kg were reinfused (range 0.25-51.9), $6.1\times10^4$ CFU-GM/kg (range 0.10-79.0) and $10.4\times10^4$ PΔ CFU-GM/kg (range 0.27-603.8). Median time to platelet count of $50\times10^9$/l was 14 days (range 9->80) and to neutrophil count of $0.5\times10^9$/l was 13 days (range 9-27). Inverse correlation was seen between the CD34+ count of reinfused cells and time to recovery of platelets (p=0.002) and neutrophils (p=0.005). Correlation was also seen between CFU-GM and platelet recovery (p=0.031) but not neutrophil recovery. There was positive correlation between CD34+ count and PΔ CFU-GM numbers (p=0.037) but no correlation was seen between PΔ CFU-GM count and engraftment time.

Conclusion: The delta assay of plastic adherent progenitor cells is capable of quantitating a primitive population in PBPC harvests but the CD34+ cell count remains superior as a means of predicting engraftment, suggesting that committed progenitors are more influential in the early phase of recovery.

### PO12 EFFECT OF AUDIT ON PRESCRIPTION OF RADIOTHERAPY FOR BRAIN METASTASES

G E Gerrard, Dr M Bottomley, H M Murphy
Yorkshire Centre for Cancer Treatment, Cookridge Hospital, Leeds.

**Introduction** The recently published Royal College of Radiologists Trial comparing 2 different fractionation schedules in the treatment of cerebral metastases concluded that 2 fractions can be as effective as 10 for the majority of patients. A reduction in the number of fractions will reduce treatment machine and radiographer workload and reduce the inconvenience to the patient. We investigated the effect of audit on prescribing practices at this centre.

**Methods** For 3 months prior to the audit meeting all patients treated with palliative radiotherapy for brain metastasis were audited. This data and the trial results were presented at an audit meeting and a departmental policy was created. Subsequently a memorandum was circulated recommending 12 Gy in 2 fractions on consecutive days for the majority of patients and 20 Gy in 5 fractions over 7 days or 30 Gy in 10 fractions over 14 days for a minority with good prognostic features. Radiotherapy fractionation for the following 3 months was again audited.

**Results** In the 3 months preceding the audit recommendations 62 patients received a total of 311 fractions of radiotherapy, giving an average of 5.0 fractions per patient. 54 patients (87.1%) received 20 Gy in 5 fractions, 4 patients received 2 fractions or less.
In the 3 months following audit 72 patients received a total of 265 fractions giving an average of 3.7 fractions per patient. 42 patients (58.3%) received 12 Gy in 2 fractions and 23 (31.9%) received 20 Gy in 5 fractions. These results indicate a 26% reduction in fractionation per patient.

**Discussion** The number of fractions used to treat brain metastases has declined in this centre in the 3 months after an audit meeting recommendation. This has resulted in some saving of limited radiographer/machine resources. Re-audit will take place in future.

### PO13

THE LEEDS TECHNIQUE, DOSIMETRY AND TOXICITY OF FRACTIONATED TOTAL BODY IRRADIATION PRIOR TO ALLOGENEIC BONE MARROW TRANSPLANTATION.

G.E.Gerrard , R.E.Taylor , G.Pitchford , D.Gilson , J.M.Povall, and A.Morgan., Cookridge Hospital , Leeds , LS16 6QB .

Introdution Since 1989 we have used a relatively straightforward technique for giving total body irradiation (TBI) , using anterior and posterior parallel opposed fields with the arms and fists acting as compensators. The dosimetry, toxicity and outcome of 48 patients treated with TBI using our technique have been audited.

Methods All patients (26 adults , 22 children) treated by this technique were followed up . The technique will be described . 14.4 Gy in 8 fractions over 4 days was prescribed to all patients with an unrelated donor and 12 Gy in 6 fractions over 3 days to those with a sibling donor. From May 1994, all children received 14.4 Gy regardless of the type of donor.

Results Lung dosimetry was - 6% to + 7% when the dose was specified to the lung maximum . The trunk doses were all within +/- 10% of the prescribed dose . Doses to other regions of the body were less homogenous but clinically acceptable in that the doses were never less than minus 10% of the prescribed dose. Thirty-eight patients developed mucositis requiring intravenous opiates. Mucositis was significantly worse after 14.4 Gy than after 12 Gy and in adults than in children. No cataracts have yet been seen. The radiation was not found to be a proven cause of clinical pneumonitis in our patients. There were no TBI related deaths.

In conclusion. our straightforward tecnique achieved reasonable dosimetry and was well tolerated.

### PO14

DIAPHRAGMATIC PARALYSIS - A NEW COMPLICATION OF CONTINUOUS 5-FLUOROURACIL?

CA O'Doherty, BR McLaren, RT Penson, ML Slevin, RTD Oliver and CJ Gallagher, , Department of Medical Oncology, St Bartholomew's Hospital, London EC1A 7BE, UK.

100 consecutive patients who had chemotherapy delivered by means of a skin-tunnelled central venous catheter were reviewed retrospectively. 92% of patients had Groshong lines inserted and 8% of patients Hickman lines. 65% of patients received chemotherapy regimens containing continuously infused 5-fluorouracil. Rates of line infection (11%), sepsis with no obvious focus (7%), line thrombosis (1%) and axillary vein thrombosis (2%) were similar to those previously reported elsewhere. However, 11% of patients receiving continuously infused 5 fluorouracil developed a previously undescribed complication of diaphragmatic paralysis on the side of the line. All of these patients had a Groshong line inserted on the right side. Clinical presentation consisted of either right shoulder pain and/or dyspnoea and diaphragmatic paralysis was confirmed with X-ray screening of the diaphragms. This problem was not seen in any patient receiving either intermittent 5-fluorouracil infusions or regimens without 5-fluorouracil. We conclude that diaphragmatic paralysis related to continuous 5-fluorouracil is a previously undescribed complication and may be due to a local irritant effect of 5-fluorouracil delivered by the side port of the Groshong line to a site adjacent to where the phrenic nerve is closely related to the subclavian vein on the right.

### PO15

RETROSPECTIVE ANALYSIS OF DOSE INTENSITY IN NEOADJUVANT CHEMOTHERAPY OF BREAST CANCER

I.Shparyk*, Dept. Oncology, POB 2468, Lviv, 290029, Ukraine.

Dose intensity (DI) in chemotherapy is defined as the amount of drug delivered per unit time and is usually standardized to body surface area as $mg/m^2/wk$. A positive relation between DI and treatment outcome has been demonstrated not only in advanced breast cancer (BC) but also in adjuvant setting. Only few trials using DI concepts have been performed in neoadjuvant chemotherapy for BC. To determine if neoadjuvant chemotherapy DI influences treatment outcome in primary BC, 44 published trials from 1985-1995 were retrospectively analyzed (4355 patient). Regimens included such agents as Cyclophosphamide or Tiotepa, Doxorubicin or Epidoxorubicin, Fluorouracil, Methotrexate, and Vincristine or Vinblastine or Vinorelbine (from single drug therapy to five-drugs combinations). Relative DI (RDI) of each study regimen was calculated against commonly used doses of each drugs in single regimens (e.g 25 $mg/m^2/wk$ for Doxorubicin, 400 $mg/m^2/wk$ for Cyclophosphamide, 25 $mg/m^2/wk$ for Methotrexate, 1250 $mg/m^2/wk$ for Fluorouracil, 1.5 $mg/m^2/wk$ for Vincristine etc.). Meta-analysis of chemotherapy trials for BC with some various regimens have suggested that higher total RDI correlated strongly with improved response rate (partial and complete response) (r=0,48; p<0,001), and complete response (r=0,52; p=0,0028). It is the first retrospective analysis on DI-response relationship (after Hryniuk e.a. in adjuvant chemotherapy and metastatic BC) in neoadjuvant chemotherapy of BC.

### PO16

VALUE OF CA15.3 IN MARKING RELAPSE IN BREAST CANCER.

T.K.Wheeler[1], S.Stenning[2], S.Negus[3], S.Picken[1] and S.M. Metcalfe[3]. [1] Dept Clinical Oncology, and [3] Dept of Surgery, Addenbrookes Hospital,Cambridge, CB2 2QQ; [2] MRC Cancer Trials Office, Cambridge CB2 2BW.

AIM. To determine any relationship between pattern of relapse and raised levels of the tumour marker CA15.3 in patients following primary therapy for breast cancer.

METHODS. Sequential patients treated for operable breast cancer and with five or more years followup were analysed for pattern of relapse. In 298 of these patients, levels of CA15.3 had been measured using the Centocor Assay kit (normal range $\leq$ 40 CA15.3 untis/ml plasma). Disease free survival curves were calculated using the Kaplan-Meier method and were compared using the log rank test. CA15.3 levels were compared using non-parametric tests because the data were not distributed normally.

RESULTS. Of the 298 patients, 66 had a clinically diagnosed relapse. The sensitivity of CA15.3 for active disease was 25/66 (37.9%) and the specificity was 231/232 (99.6%). For distant metastatic sites sensitivity and specificity was 53% and 97% respectively. Overall, the hazard rate for relapse increased upto years 4 and 5 after diagnosis for both node negative patients (upto 5%) and node positive patients (upto 15%).

CONCLUSIONS. For breast cancer patients on follow-up, a raised CA15.3 value gave a 96% probability of active disease somewhere, and 73% probability of this active disease being at a distant metastic site. Measurement of CA15.3 levels in patients at risk (node positive and > 2 years after primary diagnosis), or patients presenting with symptoms, may assist diagnosis of relapse *per se* and diagnosis of relapse at distant sites in particular.

### PO17 CHANGES IN HORMONE RECEPTORS AND PROLIFERATION MARKERS IN TAMOXIFEN TREATED BREAST CANCER PATIENTS AND THE RELATIONSHIP WITH RESPONSE.

A. Makris[1], T.J. Powles[1], D.C. Allred[2], S. Ashley[1], M.G. Ormerod[4], M. Dowsett[3]. [1]Royal Marsden Hospital, Sutton and Surrey, UK; [2]University of Texas Health Science Center, San Antonio, Texas, USA.

The primary aim of this pilot study was to establish whether there were early changes in proliferation markers and hormone receptors which could predict in individual patients, with primary breast cancer, their later response or resistance to tamoxifen. Twenty-one women with primary, operable, breast carcinomas were treated with tamoxifen 20 mg daily. Fine needle aspiration (FNA) was used to obtain samples prior to the start and at 14 days and 60 days post-treatment. From these samples estrogen receptor (ER), progesterone receptor (PgR) and Ki67 levels were determined using immunocytochemistry and ploidy and S-phase fraction (SPF) using flow cytometry. Tumour response was measured clinically according to UICC criteria. There were 12 responders (2 CR, 10 PR) and 9 non-responders (2 NC, 7 PD). Responders were more likely to be ER+ (p=0.002), PgR+ (p=0.006) and low SPF (p=0.06). At 14 days post-tamoxifen, the median decrease in Ki67 (% cells staining) for responders was -4.8 and for non-responders -0.15 (p=0.005). This decrease was seen predominantly in ER+ tumours. The difference in SPF was not significant. A decrease in ER was seen in 3/15 patients all of whom were responders. A rise in PgR was seen in 7/17 patients and all but one were responders. Similar changes for ER and PgR were seen at 60 days post-tamoxifen, however the reductions in Ki67 and SPF at that time point were not related to response.

Conclusion: We have observed a decrease in Ki67 and ER and a rise in PgR after 14 days of treatment that was related to subsequent response. These observations provide validatory support for the use of this clinical-biological model for testing the efficacy of new endocrine agents in the clinical setting.

### PO18 POLYCHEMOTHERAPY WITH INFUSIONAL 5-FU (iFU) IN LOCALLY ADVANCED BREAST CANCER - 5 YEARS' EXPERIENCE AT A SINGLE CANCER CENTER.

[1]DA Cameron, [2]A Bowman, [1]H Gabra, [2]M Stewart, [1]RCF Leonard. [1] Breast Unit & [2] ICRF Medical Oncology Unit, Western General Hospital, EDINBURGH.

**The use of iFU in metastatic breast cancer is associated with an improved response rate and altered toxicity profile. We hypothesised that the combination of iFU with other effective drugs could improve the primary medical management of inoperable breast cancer.**

**Between 1991 & 1996 we have treated 73 women for a maximum of 12 weeks with iFU in combination with other drugs ; continuous 5-FU @ 200 mg/m$^2$/day with weekly adriamycin (20 - 30 mg/m$^2$) (AcF), with three weekly cisplatin 60 mg/m$^2$ and epirubicin 50 mg/m$^2$ (ECF), or with three weekly cyclophosphamide 750 mg/m$^2$ and methotrexate 50 mg/m$^2$ (CMFinf) ; or 24 hours i5-FU @ 600 mg/m$^2$ with weekly adriamycin (20 - 30 mg/m$^2$) and cyclophosphamide 150 mg p.o. for 3 days :**

| | no. | median age | response | median survival | 2 year survival |
|---|---|---|---|---|---|
| CAF | 17 | 51 | 76% | 4.7 years | 80% |
| AcF | 33 | 50 | 76% | > 4 years | 70% |
| ECF | 14 | 46 | 79% | > 2 years | 90% |
| CMFinf | 9 | 61 | 67% | > 2.5 years | 100% |

**No significant cardio-toxicity was seen with any regimen, and the overall response rates were : pCR 4 (5%) CR 11 (15%) PR 44 (60%) SD 12 (16%) and PD 3 (4%). 46 (63 %) were operable at the end of the three months' therapy, and the median survivals were no different for those remaining inoperable or the 34 (47%) of patients with inflammatory disease at presentation. Women with ER positive tumours received tamoxifen for up to 5 years after surgery, and had a significantly better survival (p < 0.0002).**

**This retrospective analysis suggests that the use of iFU might improve the medium term survival for patients with locally advanced breast cancer.**

### PO19 IDENTIFICATION OF CLINICAL FACTORS PREDICTING OUTCOME FOLLOWING PRIMARY CHEMOTHERAPY(PMT)FOR OPERABLE BREAST CANCER

PA Ellis [1], IE Smith [1], S Ashley [1], G Walsh [1], S Ebbs [2], M Baum [1], JA McKinna [1] Breast Unit Royal Marsden Hospital, London, and Mayday University Hospital, Croydon, UK.

This study aimed to identify the clinical factors that predict for subsequent outcome in patients with large operable breast cancer treated with PMT at our institution between 1985 and December 1994. One hundred and eighty five patients (pts) received the following regimens: CMF or MMM (mitomycin, methotrexate, mitoxantrone) (76 pts); ECF (epirubicin, cisplatin, infusional 5-FU) (75 pts); AC or FEC (34 pts), followed by surgery, with radiotherapy (RT) given to those with breast conservation. Median tumour diameter was 6cm and median followup 41 months.

Overall response rate was 82% with 36% obtaining complete remission (CR). Twenty-three patients (12%) recurred locally as the first site of relapse in the absence of distant metastases. Clinical responders had improved disease-free survival (DFS)(p=0.009) and overall survival (OS)(p=0.08) compared with non-responders. There was no association between clinical or pathological CR and survival. Pre-treatment clinical axillary node positivity was a significant predictor of worsened DFS (p=0.0001), OS (p=0.0001), and local recurrence-free survival (LRFS)(p=0.03) but post-treatment pathological axillary node status did not predict for survival. Twenty-nine patients in clinical CR following PMT who electively did not have surgery and were treated with radiotherapy (RT) alone had a significantly increased rate of local recurrence compared with partial responders having surgery + RT (p=0.02). There were no differences in DFS or OS between these groups. On multivariate analysis clinical axillary node status was the only independent predictor of OS and DFS, and LRFS.

Pre-treatment clinical axillary node status is a major predictor of outcome following PCT, whereas post-treatment pathological axillary node status is not. Complete clinical response does not define a more favourable subgroup compared with those not obtaining CR. Biological predictors of clinical outcome following PMT are required.

### PO20 ALLELIC IMBALANCE AT CHROMOSOME 17P13.3(YNZ22) AND POOR PROGNOSIS IN BREAST CANCER.

A Thompson,M Clay,D Crichton,R Elton,U Chetty, C Steel. Dept of Surgery, Ninewells, Dundee, DD1 9SY.

Molecular and immunohistochemical studies of genetic events on chromosome 17p were prospectively compared with conventional clinical and pathological parameters and disease behaviour at a minimum 72 months follow up.

In a series of 91 patients with primary operable breast cancers, 37/91 (41%) patients had disease relapse and 23/91 (25%) had died during the follow up period. Allelic imbalance at the YNZ22 locus (17p13.3) examined by Southern blotting, demonstrated in 33/63 (52%) informative patients, was significantly associated with disease recurrence (p<0.01, 2df, Cox analysis) and showed a trend towards impaired survival (p=0.08, 2df, Cox analysis) after a mean follow-up of 84 months for survivors. By contrast, p53 mutation (in 10/60, 17% of cancers), p53 allelic imbalance (in 23/56, 41% informative patients), p53 mRNA expression (in 47/87, 54% patients), p53 mRNA overexpression (in 24/87, 28%) or p53 protein expression (detected in 25/76, 32%) were not associated with disease behaviour. There was no significant association between allelic imbalance at YNZ22 and any abnormality of p53 DNA, RNA or protein.

Allele imbalance at 17p13.3 (YNZ22) serves as a marker of poor prognosis in breast cancer. As yet unidentified genes on 17p13.3, distinct from p53, are therefore likely to be of clinical importance in breast cancer.

### PO21 HIGH DOSE CHEMOTHERAPY WITH AUTOLOGOUS PERIPHERAL BLOOD STEM CELL (PBSC) RESCUE FOR METASTATIC BREAST CANCER: A FIVE YEAR EXPERIENCE.

H.Gabra*[1,3], J. Craig[2], DA Cameron[3], RCF Leonard[3] [1]ICRF Medical Oncology Unit, [2]Blood Transfusion Service, Royal Infirmary, [3]Directorate of Clinical Oncology Western General Hospital, Edinburgh.

We report our experience of high dose chemotherapy with PBSC rescue between July 1992 and February 1997, representing an update of a recently published series, including more patients and longer follow-up (Cameron et al, BJC **76**, 2013-2017, 1996). Forty-six patients with metastatic breast cancer underwent 1 of 3 induction protocols. Patients with stable or responsive disease underwent high dose chemotherapy with either etoposide (1600 mg/m$^2$ ) and melphalan (140 mg/m$^2$ ) (n=10), or Thiotepa (500 mg/m$^2$) and melphalan (140 mg/m$^2$ ) (n=36). Patients were rescued with G-CSF mobilised PBSC.
Of 46 women after high dose chemotherapy, 11/46 achieved PR and 21/46 achieved CR. The only significant (WHO grade 3/4) non-haematological toxicity was mucositis. Median time to neutrophil recovery ( > 0.5 X 10$^9$/l) was 14 days, and to platelet ( > 20 X 10$^9$/l) recovery was 10 days. There were no treatment related deaths.
This approach demonstrates the feasibility and safety of this procedure and paves the way for large randomised studies

### PO22 PLANNING NEOADJUVANT CHEMOTHERAPY IN PRIMARY CARCINOMA OF THE BREAST - THE USE OF BIOLOGICAL PARAMETERS TO PREDICT HISTOLOGICAL GRADE

RK Gregory, TJ Powles, M Ormerod, J Titley, M Dowsett. Royal Marsden Hospital, London.

One of the main concerns relating to the increased use of neoadjuvant chemotherapy in primary carcinoma of the breast is that a number of patients with low grade, node negative tumours will be over treated in that they would be unlikely to receive chemotherapy in the adjuvant setting. Histological grade is an established prognostic marker and is one of the parameters upon which oncologists base decisions regarding adjuvant therapy.
**Aim**: To investigate a method for predicting histological grade on a diagnostic fine needle aspirate (FNA) in patients with primary carcinoma of the breast.
**Methods**: FNA's were obtained from patients with primary carcinoma of the breast. The sample obtained was then suspended in Eagle's essential medium. Cytospin slides were produced, one slide was sent to the cytologist for diagnostic purposes the remainder were stored at -70c and the remaining sample was snap frozen. Proliferation as measured by MIB-1 status was assessed immunocytochemically and DNA ploidy and s-phase fraction (SPF) were assessed by flow cytometry.
**Results**: In total 53 patients had a positive FNA, of these 17% were subsequently found to have grade I, 49% grade II and 34% grade III tumours. Of those patients with grade I tumours all had diploid tumours with a low SPF and MIB-1 expression (as defined by the median scores for the patient group). In contrast 89% of patients with grade III tumours had aneuploid tumours with high MIB-1 and SPF.
**Summary**: We have demonstrated that DNA ploidy, SPF and MIB-1 status can all be determined on a single diagnostic FNA and when these parameters are combined they provide an indicator of tumour grade. This may prove useful in selecting patients who may benefit from neoadjuvant chemotherapy. Follow up of patients will allow us to determine whether these pathological indices have the prognostic power of tumour grade.

### PO23 SYNERGISTIC ONCOGENES IN TRANSGENIC MICE: A REPRODUCIBLE MAMMARY CARCINOMA MODEL.

E. Tadros, K. Williamson, C. Pope, D. Kirk and W. Odling-Smee, Dept of Surgery, QUB, Institute of Clinical Science, Belfast BT12 6BJ.

Several oncogenes have been associated with human and murine mammary carcinoma. Sinn(1987) reported the development of mammary tumours in transgenic *c-myc* and *v-ha-ras* oncomice and their synergistic activity. We aimed at reproducing such a model to study the angiogenic activity of mammary tumours and angiosuppressor agents. We have successfully inbred *v-ha-ras* and *c-myc* Transgenic oncomice and crossbred them to yield dual carriers. Virgin female dual carriers developed single and multiple mammary tumours. These were observed daily from weaning age and their age at first and subsequent tumour detection was recorded. The tumour diameter was measured every 2 days and the mouse was weighed. Mice were sacrificed after 4 weeks of observation and the tumours harvested for histological identification. Mammary tumours were detected in 51 out of 109 observed mice (46.8%). This is less than 100% reported by Sinn(1987). The age at which half the mice developed tumours (T50) was 62 days ( range 43-104). Mean diameter of first tumour detected was 5.4 mm (range 3.2-8.5).Mean weight at onset of the first tumour was 23.9 grams ( 21.8-25.8). More than half developed single tumours. Histologically these tumours were adenocarcinomas with a papillary component.

| No of tumours/mouse | 1 | 2 | 3 | 4 | 5 | 6 | Total |
|---|---|---|---|---|---|---|---|
| No of mice | 33 | 5 | 4 | 1 | 3 | 5 | 51 |
| % of group/total | 64.7 | 9.8 | 7.8 | 2.0 | 5.9 | 9.8 | 100 |

Table 1 shows the incidence of multiple tumours.

### PO24 NAVELBINE (NVB) & FRACTIONATED DOSE DOXORUBICIN (DX) IMPROVES 1ST LINE ADVANCED BREAST CANCER (ABC). AN OVERVIEW OF 3 PHASE II TRIALS.

J. Carmichael[1], R. Hegg[2], D. Firat[3], M. Pawlicki[4], F. Le Bras[5], F. M. Delgado[5], P. Danel[5] and S.A.N. Johnson[6]. [1]Nottingham City Hosp UK; [2]ELAN Brazil; [3]Ankara, Turkey; [4] Krakow-Poland; [5]I.R.P.F., France; [6]Taunton & Somerset Hosp, UK

**Aim**.-Anthracycline combinations represent the most powerful chemotherapeutic approach in the treatment of ABC, but their limiting toxicities are neutropenia and cardiac impairment. NVB as a single agent has demonstrated a high activity and good tolerance in ABC: 40%-60% response rate (RR). Promising results have previously been obtained with NVB 25 mg/m² D1 & 8 + DX 50 mg/m² D1 (q 3 w): 74% RR (21% CRs), mainly in visceral sites (JCO 94). This was confirmed by a significant survival advantage observed in pts with liver metastases treated with NVB + DX compared to CAF (ESMO 96). Dividing the DX dose and administering it at weekly intervals may reduce the cardiotoxicity without substantially impairing the efficacy. 3 studies were conducted with NVB + DX, both at 25 mg/m² D1 & 8 (q 3 w, 8 cycles) to check the efficacy, improve the tolerance and to ease outpatient administration. **Results**.- 120 pts were included: age 30-73y; PS 0-1: 85%; visceral involvement: 52%; adjuvant C: 18%. 668 cycles were administered; WHO grade (G) 3-4 neutropenia: 24%; infection G 3: 6/120 pts; G 3-4 nausea/vomiting: 17 pts; G 3-4 constipation 1.5%; G 1 peripheral neuropathy: 13%; G 3 alopecia: 53.5%. No G 3-4 cardiotoxicity. The RR ranges from 70% to 77% (18-35% CRs) RR on visceral sites: 56%-86%. **Conclusion**.- These results confirmed that NVB + DX (25 mg/m² D 1 & 8) has major and reliable activity as 1st line therapy. Given its very favourable tolerance, low morbidity and absence of life threatening cardiotoxicity, out patient administration of this regimen could be recommended as 1st line treatment for ABC.

### PO25 USAGE OF AN INTERNET-BASED CANCER INFORMATION SERVICE FOR THE GENERAL PUBLIC (CANCERHELP UK) VIA THE INTERNET AND IN CLINICAL SETTINGS.

S J Tweddle[1], C James,[1] D Davies,[2] P Harvey,[3] L Woolf[4], N James.[1] [1]CRC Institute for Cancer Studies, The University of Birmingham, Edgbaston, Birmingham B15 2TA, [2]School of Medicine & Dentristry, The University of Birmingham, Birmingham, B15 2TJ, [3]Birmingham Oncology Centre, Queen Elizabeth Hospital, Edgbaston, Birmingham, B15 2TH, [4]BACUP, 3 Bath Place, London, EC2A 3JR.

An Internet-based cancer information service for the general public, CancerHelp UK, is being evaluated through usage in clinical settings and on the Internet.

In the study of usage in clinical settings data are gathered through pre- and post- use questionnaires, interviews and observation of patients and carers in 2 hospital clinics and 3 GP surgeries; participants are asked about information needs and information media preferences. Post-use questionnaires ask about the ease of use, design and usefulness of the service.

The study of Internet usage compares data from two samples of users over two four month periods. Feedback forms completed by Internet users and user activity recorded on the computer server generate data about users, their evaluation of the service and usage patterns.

80% of the participants in clinical settings had only occasionally or never used a computer; 92.3% had rarely or never used the World Wide Web; 22.1% declined to use CancerHelp UK altogether. 82% of the first Internet sample (89) respondents were female and friends and relatives were the largest group of respondents (36%). Initial findings show that users are positive, find the service easy to use and are happy with existing information but wish for additional detailed information on treatment and specific cancers.

### PO26 COULD THE ROTTERDAM SYMPTOM CHECKLIST (RSCL) BE USED IN THE ROUTINE CARE OF PATIENTS WITH CANCER? A PURCHASER-PROVIDER COLLABORATION.

P Revell[1], JP Walsworth-Bell[2], MS Young-Min[1], S Wardle[2], JM Anslow[1].
[1]Dept Haematology, Staffordshire General Hospital, Stafford ST16 3SA,
[2]Dept Public Health Medicine, South StaffordshireHealth Authority Stafford, ST16 3SR.

In discussions around cancer treatment services in 1994 it became apparent that much cytotoxic chemotherapy is for palliation, not cure. Quality of life issues therefore become important in purchasing decisions and the patient's own perception is perhaps the most relevant.

A 2 year pilot study was initiated to see if substantial numbers of cancer patients could provide on-going quality of life data using a validated instrument and with minimal disruption to themselves and the clinic - using current structures rather than new resources. Patients in the haematology clinic with CLL and LG-NHL were chosen as chemotherapy is palliative but with a low number of expected deaths in the study period (observed = 5/68).

A reformatted RSCL (2 sides of a single A4 sheet) was sent by post, with a personal covering letter, in advance of the next scheduled clinic visit. The patient was asked to assist us by filling in the form at home on the day before the clinic and hand it in when attending. A maximum of 4 episodes were requested at a minimal interval of 3 months.

184/207 (89%) of forms were returned first time; 51/68 (75%) of patients returned forms for all their episodes first time. All non-responders "caught up" with repeat forms sent for subsequent visits.

Anonymised forms were sent to the HA for analysis. The instrument appears valid in this context though the "work" and sexual activity modules seemed less relevant to this (generally older) group of patients - removal of these 2 aspects did not meaningfully alter median scores.

It became apparent during the pilot that although participation prompted patients into discussing subjects in the clinic they might otherwise not have mentioned, it was of limited real-time usefulness. A further study is planned to see whether the checklists can be expeditiously scored and the information used in the clinic. Could "routine" follow up of cancer patients ultimately be replaced by targeted clinic attendances based on postal quality of life data?

### PO27 PATIENT HELD RECORD

M Buckley. I G Finlay.
Velindre NHS Trust, Cardiff, Wales, UK

**Object.** Following a survey held with general practitioners (GP's) and patients (1994) the author identified the need to develop a patient held record (PHR) for cancer patients.The aim of such a record was to help improve communication between the hospital,GP's and primary health care team. The objective of the pilot was to identify if such a record would be useful in improving communication between the hospital and the community services.

**Method.** A working group, which included GPs compiled a draft patient held record. The record was piloted for 4 months on 100 out-patients. The 100 patients were individually interviewed with a relative or a carer and were given verbal and written guidelines in the aim and use of the record.

**Results.** After 4 months of the pilot the first level analysis was performed. The data collection tools were questionnaires designed for the 100 patients and 100 health care professions. Overall response rate = 83.5%. The results suggest that the PHR would be a useful aid in improving communication between services when used correctly. Patients found the record acceptable and valued the way in which they were consulted about what was being recorded.Patients and doctors had different attitudes and expectations of the patient held records. GP's looked at it as a management and communication tool, whilst patients mostly perceived the patient held record as a personal document for reference, safety and to be able to "keep in control". Hospital staff failed to enter details in the record, did not readily hand out PHRs to new patients and did not encourage patients to participate in their core planning documentation.

**Conclusion.** The pilot shows that PHRs are welcomed by patients, GP's and other agencies. However resistance to change was evident amongst hospital staff. The obstacle from relates to approach, attitudes, perceptions and anxieties of these health care professionals, further education and training is required.

### PO28 PULSED FIELD GEL ELECTROPHORESIS IN NORMAL TISSUE RADIOSENSITIVITY TESTING.

**AE Kiltie, A Ryan, CJ Orton, CML West, JH Hendry. Department of Experimental Radiation Oncology, Paterson Institute for Cancer Research, Christie Hospital NHS Trust, Manchester M20 4BX.**

**Using clonogenic assays, fibroblast survival has been found to correlate with radiotherapy late normal tissue reactions, but such assays take too long to be of use in predictive testing of individual patients. It has been shown that residual DNA damage in fibroblasts as measured by pulsed field gel electrophoresis correlates with clonogenic survival, when radiosensitive ataxia-telangiectasia lines are included. We have undertaken a study which confirms this correlation using fibroblasts cultured from nine pre-therapy cervix cancer patients ($SF_2$ values ranging from 0.147 to 0.322). Tritium-labelled cells were irradiated in plateau phase and allowed to repair for 24 hours, before cell-agarose plugs were made and lysed prior to PFGE. The residual damage slope (from 60-150Gy) correlated with Dbar both when two radiosensitive cell lines were included ($r=-0.91$, $p<0.001$) and for the vaginal fibroblasts alone ($r=-0.83$, $p=0.006$). The fraction of activity released (FAR) at 150Gy correlated with residual damage slope ($r=-0.92$, $p<0.001$), suggesting that a single dose point may be used in studying patient material.**

**Primary biopsy material would allow a more rapid assay using PFGE, but cells cannot be pre-labelled. We have developed a simple method of gel imaging, using relatively inexpensive CCD TV equipment and commercially available computer software to analyse SYBR green stained gels, which is as sensitive as radioactive prelabelling.**

### PO29

### FUNCTIONAL ASSAY OF Pgp ACTIVITY IN SOLID TUMOR SPECIMENS EX VIVO,

T. Bogush, G. Smirnova, E. Chmutin, Cancer Research Center, Moscow, Russia

It is generally accepted that the results of direct immunohistochemical determination of Pgp expression poorly correlate with the chemotherapy outcome and that a functional assay is necessary for assessment of Pgp function. The commonly used functional assays of Pgp activity are applied to cell suspensions only, and no functional intravital assay is available for investigation of solid tumors. We have developed a functional assay of Pgp activity in solid tumor specimens based on assessment of Pgp response to Pgp modifiers. The measured parameter is the intracellular accumulation of doxorubicin (DOX) which is Pgp-dependent. To measure DOX intracellular accumulation in solid tumor specimens, we have adapted the previously developed fluorimetric approach which was designed for measurement of doxorubicin (DOX) intracellular transport and binding to DNA in cell suspensions. The method is based on the phenomenon of DOX fluorescence quenching after interaction of the drug with DNA but not with other cellular macromolecules. When a solid tumor specimen is incubated with DOX, the fluorescence of the drug in the incubation medium decreases reflecting both intratumor penetration and intracellular accumulation. After reaching the stable rate of fluorescence quenching a Pgp modifier is added to the incubation medium and DOX fluorescence quenching is further monitored. If the modifier acts on Pgp, the increase of the speed of DOX fluorescence quenching in incubation medium occurs. Using this methodology we have shown the Pgp functional activity in solid murine resistant Ehrlich tumor and the absence of such activity in the sensitive one. We now apply the developed methodology for investigation of human biopsy solid tumors to reveal the relationship between the tumor Pgp functional activity and response to chemotherapy. Supported by the Russian Foundation for Basic Researches.

### PO30

### A QUANTITATIVE CELLULAR (MURINE) MODEL OF CYTOTOXIC ORAL MUCOSITIS.

AM WARDLEY, JH SCARFFE, CS POTTEN.

CRC Department of Epithelial Biology, Christie Hospital & Paterson Institute, Manchester M20 4BX, UK

Oral mucositis (OM) is a major and often dose-limiting side-effect of cytotoxic agents. Manipulation, with cytokines, of stem cell kinetics to render them more resistant to damage before treatment and subsequently stimulate them to proliferative more rapidly after therapy may abrogate this toxicity. The investigation of such agents requires a quantitative cellular model.

$BDF_1$-mice were given OM-inducing therapy (head-irradiation and/or chemotherapy with 5-fluorouracil, melphalan or bleomycin). Animals were sacrificed daily after being pulsed with bromodeoxyuridine. Using a Zeiss Axiohome TM computer assisted microscope cells/$mm^2$ epithelium and cells/mm basement membrane were counted for ventral tongue and buccal mucosa. The basal labelling-index was calculated. There is a progressive decrease in cellularity to reach a dose dependent nadir followed by rapid repopulation to baseline levels. Complete suppression of DNA synthesis, is followed by a progressive regenerative increase with a maximum coincident with cellular nadir. This in vivo model describes the changes in epithelial cellularity which cause OM and will be used to investigate the potential of various agents to protect against or accelerate recovery from therapy induced mucositis.

### PO31

### LASER INDUCED HYPERAEMIA- HOW MUCH, WHEN AND WHY?

A R Gillams, A Waldman, S G Bown,* G Buonaccorsi* and WR Lees
The Departments of Medical Imaging and The National Laser Centre,* The Middlesex Hospital, London, UK.

**Object:** To study the effect of interstitial laser therapy on perfusion in patients with liver tumours.

**Methods:** Twelve patients who had been referred for interstitial laser therapy were studied with dynamic helical contrast CT both before and after treatment. 5/12 were restudied at 2-3 months follow-up. Following a bolus of intravenous contrast a single axial CT slice was performed through the lesion at 1.1 second intervals. Scanning commenced at 12 seconds after the onset of the injection and continued for 33 seconds. Region of interest measurements were made over the aorta, portal vein, adjacent liver and distant normal liver.

**Results:** Following laser therapy an area of hyperaemia, seen as a focal increase in contrast enhancement, develops around the lesion. Two patterns were identified: circumferential or involvement of the whole tumour bearing segment. Up to a four fold increase in hepatic arterial flow was observed at 12 hours post treatment. There were milder increases in portal venous flow. This effect persisted but was less marked at 3 months follow up.

**Conclusion:** Laser therapy induces a marked increase in hepatic arterial perfusion around tumours. It is possible that this effect could be used to improve delivery of chemotherapeutic agents specifically to the tumour.

### PO32

### DIFFERING PATTERN OF IN VITRO CEA EXPRESSION AND SUPERNATANT RELEASE BY AUGMENTED COLORECTAL CANCER CELLS

M.H.S. COLLIE; J. BHATIA; R. AUSTEN; M. C WINSLET, Dept of Surgery, Royal Free Hospital, Pond St, London NW12.

Colorectal cancer cells are known to be heterogenous expressors of the tumour associated antigen Carcinoembryonic Antigen (CEA), which may be targetted by antibodies. The efficiency of this antibody guided detection and treatment is compromised by the heterogeneity of expression of the CEA.

Certain chemical agents, cytotoxic drugs and environmental changes have been shown to increase CEA expression in colorectal cancer cells.

In this study, three colorectal cancer cell lines, Lovo,HT29 and Colo, known to be high, low and non-expressors of CEA were grown in monolayer cell culture for five days in the presence of a differentiating agent (Butyric Acid, Theophylline, 5-Azactidine or Interferon), or with altered environment (Acid medium, alkaline medium, starvation or hypoxia)or with 5Fluorouracil. The cells were then immunostained and their CEA expression measured by Fluorescein Activated Cell Sorting. Radioimmunoassay of the supernatants from the culture flasks was performed to measure CEA released.

Significant changes in CEA expression and release as analysed by the Wilcoxon Rank Sum test ($P<0.05$) were as follows:

| | Starve | Acid | Alkali | Hypox | ButAc | Theop | 5-Azac | IFN | 5FU |
|---|---|---|---|---|---|---|---|---|---|
| Colo | | | | Exp ↑ | | | Exp ↑ | | |
| HT29 | Rel ↑ | Exp ↑ | | Exp ↑ | Exp ↑ | Exp ↑ | Exp ↑ | Exp ↑ | Exp ↑ Rel ↑ |
| Lovo | Exp ↓ Rel ↓ | Exp ↓ Rel ↓ | Exp ↓ Rel ↓ | | | Rel ↓ | Exp ↓ Rel ↓ | Exp ↑ Rel ↓ | |

The changes in CEA expression were not mirrored by similar or reciprocal changes in supernatant release, although results were influenced by natural expression levels.

In vitro, it is concluded that chemical agents or environmental changes which lead to augmented CEA expression by colorectal cancer cells do not cause matching increases in CEA supernatant release. If the same mechanism applies in vivo, augmentation may not necessarily increase serum CEA levels, with resultant high non-specific antibody binding and background noise.

### PO32B

**GROSS VARIATION IN CEA EXPRESSION AUGMENTATION BY DIFFERENTIATING AGENTS**

M.H.S.COLLIE, N.J.BRADLEY, M.C.WINSLET, Dept of Surgery, Royal Free Hospital, Pond St., London NW12.

Colorectal cancer cells are heterogenous expressors of the tumour associated antigen Carcinoembryonic Antigen (CEA). It is possible to target CEA on tumour cells in vivo with anti-CEA antibodies, conjugated to radioisotopes or cytotoxic drugs for the purposes of detection and treatment. The efficiency of this antibody guided detection is compromised by the heterogeneity of expression of the CEA.

This study compared the effects of 4 differentiating agents, (Butyric Acid, γ-Interferon, Theophylline and 5-Azacytidine) on 3 colorectal cancer cell lines, Lovo, HT29 and Colo, known to be high, low and non- expressors of CEA respectively. The cells were grown in standard tissue culture with one of the agents for 5 days, before being harvested and immunostained for CEA. The degree of CEA expression was analysed by Fluorescein Activated Cell Sorting.

Average Fluorescence of Cells:

| | Control | But Acid | Interferon | Theoph | 5-Azacyt |
|---|---|---|---|---|---|
| LOVO | 544.0 ± 156.3 | 768.2 ± 185.6 | 807.4 ± 45.2 | 575.07 ±117.8 | 238.6 ± 28.3 |
| HT29 | 18.3 ± 2.6 | 45.2 ± 5.4 | 33.1 ± 3.2 | 30.9 ± 2.4 | 29.4 ± 6.0 |
| COLO | 9.8 ± 7.2 | 11.5 ± 2.2 | 13.2 ± 4.7 | 61.9 ± 35.9 | 78.6 ± 35.3 |

Different colorectal cancer cell lines may be induced by various differentiating agents to increase their expression of CEA, but they do not appear to share common pathways to CEA expression.
Using a combination of agents may be a more efficacious way of inducing the increased CEA expression of a typical heterogenous colorectal cancer.

### PO33

**PROTOPORPHYRIN IX FLUORESCENCE IN THE DIAGNOSIS OF DYSPLASIA IN BARRETT'S OESOPHAGUS**

M A Jahan, I S Tait, E L Newman, A Cuschieri,
Department of Surgery, Ninewells Hospital, Dundee,

*Background and aims*-The vast majority of oesophageal adenocarcinoma cancers arise on a background of Barrett's metaplasia.The increased risk of malignant transformation in Barrett's is between 40 -150 %. Patients with Barrett's oesophagus are followed up by endoscopic surveillance to detect dysplastic and malignant change. The current practice of taking multiple random biopsies has a poor sensitivity and specificity in detecting high grade dysplasia and early adenocarcinoma. We have studied delta-aminolaevulinic acid (ALA) induced protoporphyrin IX (PpIX) fluorescence as a marker to target endoscopic biopsies. *Methods*-20mg/kg of ALA dissolved in orange juice were administered orally 4-6 h before endoscopy to 40 patients with Barrett's oesophagus and 4 with oesophageal cancer. PpIX fluorescence was excited by a non-laser blue light source. Biopsies were taken from both fluorescent and non-fluorescent areas and submitted for histology. *Results*-70 biopsies were analysed histologically and compared against fluorescent and white light endoscopy.The sensitivity and specificity of diagnosing dysplasia in Barrett's and cancer using fluorescence were 35.7% and 77% respectively compared to 28.5% and 85.5% for conventional white light endoscopy. These differences did not reach statistical significance. Side effects were minimal, one patient withdrew from the study, two others developed mild cutaneous sensitivity. Four developed mildly elevated liver enzymes which resolved within 48 hours. *Discussion*-Sensitivity was improved by the use of fluorescence to guide biopsies, though the effect was small. Current efforts are focused on better matching of the light source with the absorption peak of PpIX efficient delivery of light via an ultraviolet fibre. We expect improved fluorescence excitation and enhanced sensitivity with the new apparatus.

### PO34

**A PHASE II STUDY OF INTRAVENOUS GAMMALINOLENIC ACID (GLA) PRE-TREATMENT IN PATIENTS WITH PANCREATIC OR COLORECTAL CANCER TREATED WITH 5-FLUOROURACIL (5-FU).**

R Bryce[2], JA Ledermann[1], I Taylor[1], C Brennan[1], RCG Russell[1], A Hatfield[1], [1]University College London Hospitals, London, UK, [2]Scotia Pharmaceuticals Ltd, Stirling, UK.

GLA is cytotoxic to pancreatic cancer cell lines and synergism has been shown when it is added to conventional cytotoxics in vitro. Twenty patients with histologically confirmed advanced unresectable pancreatic (18) or colorectal (2) carcinoma have been treated with intravenous GLA 0.84g/kg (0.28g/kg for the last 5 patients to reduce haemolysis and administration time) followed by 12 weeks' continuous infusion with 5-FU 300mg/m$^2$ week. Patients had Karnofsky Performance Status >60% and gave written informed consent. Two are still receiving 5-FU. Four completed 5-FU as scheduled, 4 completed but required dose reduction or interruption due to toxicity, 5 withdrew because of 5-FU toxicity or Hickman line problems and 5 died before completing 12 weeks. Grade 4 toxicities were mucositis (1), planar-palmar erythema (1), vomiting (1). Four patients still survive, the longest lived for 389 days to date. Kaplan Meier analysis of survival times yields a median of 201 days for all patients, and 200 days when restricted to patients with pancreatic cancer. The data from this study suggests GLA does not result in any significant increase in clinical toxicity of 5-FU and may be a useful adjunct to conventional chemotherapy in advanced pancreatic cancer.

### PO35

**TUMOUR LOCALISATION OF AN ANTI-CEA SINGLE CHAIN FV ANTIBODY IN COLORECTAL CANCER**

A. Mayer[1], G.Boxer[1], D. O'Malley[1], K. Chester[1], B. Davidson[2], M.C. Winslet[2], A.J.W. Hilson[3], R.H.J. Begent[1]. ([1]CRC Targeting and Imaging Group on behalf of the phase I/II committee, Dep. of Clin. Onc., [2]Dep. of Surgery, [3]Dep. of Med. Physics, Royal Free Hospital MS, London)

Patients undergoing resection of primary or recurrent colorectal cancer are enrolled into a phase I study in order to investigate the potential of Radioimmunoguided Surgery (RIGS®) to localise tumour deposits using an anti-CEA single chain Fv (scFv) antibody. ScFvs consist of the variable heavy and variable light chain region tethered by a flexible linker and are as such the smallest antibody fragments to retain full binding capacity.

Patients receive $^{125}$I labelled MFE-23-his, an scFv antibody derived from bacteriophage technology with high affinity for CEA (Chester et al, 1994) 24, 48 or 72 hours prior to operation. The abdomen is scanned after traditional exploration with a hand-held gamma-detecting probe (Neoprobe®1000 instrument). The endpoint of the study is the correlation of results of the probe with histology and counts of the resected specimen obtained by a laboratory gamma counter.

Preliminary results show selective tumour localisation in 2 patients undergoing resection of liver metastases 72 hours after injection of the antibody. RIGS findings correlated with histology in 5 patients with primary tumours resected 24 (4 patients) or 48 hours (1 patient) after injection. Due to the clearance of the antibody higher tumour to blood and tumour to normal tissue ratios were found 72 hours after injection, namely 13:1 and 4.8:1, allowing improved discrimination of tumour and normal tissue.

These results show that scFv can be successfully prepared and used for RIGS procedures. Further improvements of the technique should allow detection of small volume disease and therefore improve selection of patients for adjuvant treatment, particulary antibody-targeted therapy.

### PO36 THE EFFECTS OF UROGRAFIN ON THE PARTICLE SIZE AND THE UPTAKE OF LIPIODOL IN TARGETED THERAPY FOR HEPATOCELLULAR CARCINOMA

R.A.M. Al- Mufti, L Tuwau, K.E.F. Hobbs, M.C. Winslet. University Department of surgery, Royal Free Hospital School of Medicine, Pond Street, London, NW3 2QG.

Lipiodol, an iodinated ethyl ester derivative of poppy seed oil, has been used in targeted therapy for primary and some metastatic hepatic cancers. Lipiodol is insoluble in water, and Urografin (diatrizoate) is used clinically to emulsify Lipiodol prior to its intra-arterial injection. The effect of Urografin in combination with Lipiodol was assessed in tissue cultures of Hep-G2 (hepatoma) cell line. The cell cultures were exposed to 2% Lipiodol alone and 2% of Lipiodol with 2% Urografin for variable duration (3, 6, 12, 24, 48 and 72 hours). The uptake and retention of Lipiodol was assessed using computer-assisted image analysis. The size of the Lipiodol particles were measured after 2 minutes agitation of the oil suspensions with culture media.

**Mean Number of Particles per field [n=4] (+/- SD)**

| | < 10 μ | 10-25 μ | 25-50 μ | 50-100 μ | >100 μ | Total |
|---|---|---|---|---|---|---|
| 2% Lipiodol | 890 (101) | 110 (38) | 17 (3) | 4 (1) | 4 (1) | 1,025 |
| 2% Lipiodol + Urografin | 575 (73) | 220 (56) | 21(4) | 7 (2) | 8 (2) | 831 |

The mean size of the Lipiodol particles when combined with Urografin was considerably larger than that of Lipiodol alone, with a reciprocal reduction in the number of Particles.

**Quantification of Lipiodol uptake by computer assisted image analysis (Optical Density / computerised arbitrary units)**

| | 3 hours | 6 hours | 12 hours | 24 hours | 48 hours | 72 hours |
|---|---|---|---|---|---|---|
| 2% Lipiodol | 52.2 | 91.4 | 92.4 | 131.2 | 139 | 142 |
| 2% Lip. + Urografin | 49.4 | 59.8 | 68.8 | 89.8 | 115.4 | 128 |

The addition of Urografin to Lipiodol resulted in a significant delay in the uptake of Lipiodol by the cancer cells in vitro. A similar phenomenon in vivo may interfere with the delivery of Lipiodol-targeted therapy for these tumours. The continued use of Urografin to dissolve Lipiodol in clinical practice requires further evaluation.

### PO37 OESOPHAGEAL STENT INSERTION FOR INOPERABLE CA OESOPHAGUS. IS IT OF VALUE?

A.Robinson[1], A.Solieri[2], J.O'Brien[3], G.Bray[4], C.Trask[5], A.Lamont[6], [156]Dept. Oncology, [2]Audit, [34] Dept. Gastroenterology, Southend Hospital, Prittlewell Chase, Westcliff, Essex, SS0 0RY

Retrospective review of oesophageal stent insertions from 01.08.95 - 31.12.96 was carried out. Objectives:

1. Are patients selected appropriately? (Defined survival >2/12)
2. Is relief of dysphagia achieved?
3. Are Dietitian and Macmillan services enlisted?
4. Is information about procedure given?

19 patients, histologically proven (47.3% Adeno., 47.3% SCC and 5.3% Small Cell) with inoperable Ca oesophagus, were stented using covered oesophageal stents. 57.9% female, mean age 70.4 years. All had significant weight loss (10% body weight) and 20% total dysphagia at time of procedure. Procedure was well tolerated, one (5.2%) complication - cardiac arrest (patient successfully resuscitated, subsequently underwent stenting). 52.6% received additional XRT, 10.5% chemotherapy and 36.8% no further treatment. 63.1% survived >2 months post procedure, mean survival 7 months, all tolerated semi-liquid to normal diet up to death. 80% and 74% had Dietitian and Macmillan nurse involvement respectively. An information leaflet was given to 74% patients.

Conclusions: Stenting achieved significant palliation. Now standard procedure to refer patients to Dietitian, multidisciplinary meetings ensure access to Macmillan nurse when appropriate.

### PO38 SURGERY PLUS CHEMOTHERAPY VS CHEMOTHERAPY ALONE FOR PRIMARY INTERMEDIATE- AND HIGH-GRADE GASTRIC NHL

R.A. Popescu[1], A.Wotherspoon[2] and D.Cunningham[1] *The Royal Marsden NHS Trust, Sutton, Surrey, Departments of [1]Medicine and [2]Histopathology*

Primary gastric lymphomas (PGL) have traditionally been treated with surgery followed by chemotherapy or radiotherapy. Surgery was thought to improve staging, optimise local disease control and reduce risk of perforation or bleeding, but its role has recently been questioned given improved CT and endoluminal USS staging, and efficient chemotherapy. We used a prospective database to identify patients with PGL (defined according to criteria by Lewin and Herrmann) treated at the RMH since 1985 with surgery followed by chemotherapy (n=13) or chemotherapy alone (n=25). A larger proportion of patients in the chemotherapy alone group had anorexia, abdominal pain, a palpable epigastric mass or GI bleeding at presentation. Equally, more patients had advanced disease on Mushoff staging. All lymphoma-related deaths occurred in the first year following diagnosis. Overall survival was identical in the surgery plus chemotherapy and the chemotherapy alone group, ( 87.6 vs. 84.6 %), and the major determinator of outcome was stage at diagnosis ( Stage IE and IIE1 100% 5 y survival, more advanced stages 76 % 5 y survival). 3 of 13 patients developed malabsorption after gastrectomy, while 5/25 patients receiving chemotherapy alone had GI bleeding. 2 patients had extensive inoperable tumors invading the whole gastric wall, and 1 patient had recurrent bleeding and underwent gastrectomy. No perforations took place. In this series, chemotherapy alone was as efficient as the combination of surgery and chemotherapy in intermediate and high-grade PGL of any stage, and no perforations or serious bleeding occurred.

### PO39 HEPATIC ARTERIAL FLOXURIDINE IN SYSTEMIC 5FU / FOLINIC ACID RESISTANT COLORECTAL LIVER METASTASES

C. Fordy, C. Glover, K. Handscomb, M. Davies, T.G. Allen-Mersh. Dept. of Surgery, Charing Cross and Westminster Medical School, Chelsea and Westminster Hospital, London, SW10 9NH.

The management of colorectal liver metastasis patients (CLM) who have failed to respond to systemic 5 Fluorouracil (5FU) / Folinic Acid is unclear. Hepatic arterial infusion (HAI) of Floxuridine (FUDR) has been reported as producing tumour response in comparative studies where non-responsive systemic patients were "crossed over" to HAI.

We have assessed response and response duration in 33 patients (median tumour volume 322 mls, IQR 55 mls, 736.75 mls) with CLM progression on systemic chemotherapy treated with a median of 6 cycles of continuous HAI Floxuridine 0.2mgs / kg / 24 hours over 14 days.

Partial response (>50% tumour shrinkage) occurred in 3 (10%) and disease stabilisation (any tumour shrinkage) in a further 4 patients. Duration of disease stabilisation (interval to metastasis regrowth above baseline CT scan) was 9.5 months. Fall in serum CEA occurred in 22 (67%) patients, the duration of serum CEA fall was a median of 6 months (IQR 4.25, 12).

193 treatment courses were delivered and 48 treatments were omitted because of toxicity. WHO grade 1 or 2 toxicity (nausea / vomiting, stomatitis, gastritis or diarrhoea) occurred in 11 (33%) and reached grade 3 or 4 in 10 (30%) patients.

HAI Floxuridine in systemic 5FU resistant patients stabilised liver metastases in 21% of patients for 6 - 9 months. The results are similar to other reported second line chemotherapy regimens in advanced colorectal cancer. HAI may offer benefit to selected CLM patients with systemic 5FU resistant disease.

### PO40

INTRA-LUMINAL BRACHYTHERAPY FOR CHOLANGIOCARCINOMA USING THE NEW ULTRAFLEXIBLE "MICROSELECTRON-HDR V2" OBLITERATES THE NEED FOR AFTERLOADED IRIDIUM.

P.B. Rogers[1], A.J. Vinall[2], J. Solano[1], A. Hatfield[3], M.F. Spittle[1]. Departments of [1]Clinical Oncology, [2]Medical Physics and [3]Gastroenterology, Middlesex Hospital, London W1N 8AA, UK.

INTRODUCTION: The Middlesex Hospital is a clinical trial site for the new *microSelectron-HDR V2*, made by Nucletron. Since 1989 we have tried to use the *"Classic microSelectron"* in 40 patients to give intra-luminal brachytherapy via a nasobiliary tube placed at ERCP. The cable has only been flexible enough to treat one patient. The source was unable to pass the acute duodeno-choledochal angle (DCA) in 98% patients who therefore required treatment with manually afterloaded Iridium, necessitating admission for one week to undergo ERCP and brachytherapy over 2-3 days in the radiation protection room. Patients also received external beam radiotherapy.

OBJECTIVE: To assess whether the new *microSelectron-HDR V2* can negotiate the most acute DCA to treat inoperable cholangiocarcinoma.

METHOD: The *microSelectron-HDR V2*, with a 20% smaller source size (0.9mm diameter) and more flexible cable, was tested on 2 patients with acute DCA's. During ERCP the 1.5m microselectron catheter was placed in position. A dummy source was used to assess feasibility prior to treating the patient.

RESULT: The dummy source made three clear test passes. The remote afterloading high dose rate Iridium source was therefore used to treat the cholangiocarcinoma. A dose of 18 Gy to 0.5 cm was given over 3 minutes and 19 seconds, thereby treating a patient who would otherwise have required treatment with a manually afterloaded source as an inpatient over 3 days.

CONCLUSIONS: The new machine has important advantages:

1. It will eliminate the need for inpatient care in the radiation protection room, allowing the patient home on the same day.
2. It will decrease radiation exposure to physicists, doctors using the manually afterloaded Iridium, nursing staff and visitors.
3. It will allow the radiation protection room to be used for other treatments thereby decreasing waiting times.
4. It saves the cost of the use of a bed for one week.

### PO42

SUOG/SCTN NATIONAL SCOTTISH PROSTATE CANCER AUDIT,

Windsor PM[1], Thomson CS[2], Stroner PL[3], Goodman C[4]. [1] Department of Radiotherapy, Ninewells Hospital, Dundee DD1 9SY, [2] Scottish Cancer Intelligence Unit, ISD Scotland, Trinity Park House, South Trinity Road, Edinburgh EH5 3SQ, [3] Scottish Cancer Therapy Network, ISD Scotland, Trinity Park House, South Trinity Road, Edinburgh EH5 3SQ, [4] Department of Urology, Dundee Royal Infirmary, Dundee.

All men newly diagnosed as having prostate cancer were identified from the Scottish Cancer Registry, 1221 men in 1988 and 1591 men in 1993. Hospital records were accessed by Scottish Cancer Therapy Network data managers and an audit data set extracted, 91% of notes were accessed. Tumour staging was often poorly documented or omitted. Staging bone scans were carried out in only 43% of patients in 1988 rising to 58% in 1993, with an increase in negative scans. There was a rise in radical radiotherapy from 67 (5.5%) in 1988 to 134 (8.4%) in 1993; this showed a marked regional variation, most referrals (74% and 64%) were by urologists. Most patients having radical radiotherapy had undergone TURP (76% and 66%), the use of biopsy increased (21% rising to 33% in 1993). Overall only 5.8% of patients in 1988 and 10.4% in 1993 had radical (curative) treatment by radiotherapy or radical prostatectomy. As a percentage of documented M0 patients (244 and 479 respectively) this amounted to only 29% in 1988 rising to 34% in 1993.

### PO41

**A PHASE II STUDY OF INTERFERON alpha-2a (IFNα) AND MODULATED 5-FLUOROURACIL (5-FU) IN PATIENTS (pts) WITH ADVANCED NEUROENDOCRINE TUMOURS (NET).**

D.Papamichael, R.T.Penson, M.T.Seymour, P.Wilson, C.J.Gallagher, G.M.Besser, M.L.Slevin.

***Background***: Both 5FU and IFNα have shown modest single agent activity in pts with NET, with biochemical responses much more common than objective responses. The combination of 5FU and IFNα has shown synergy in tumour models, and activity in several gastrointestinal malignancies.

***Methods***: Fifteen pts with advanced NET (12 carcinoid, 2 islet cell, 1 phaeochromocytoma) of whom 3 were non-secretors, were treated with leucovorin 200mg/m$^2$ intravenous(i.v.) infusion over 2 hours, then 5FU 400mg/m$^2$ i.v. bolus followed by 400mg/m$^2$ i.v. infusion over 22 hours, all repeated on day 2; IFNα was given at 6 x 10$^6$ IU subcutaneously every 48 hours throughout. Treatment was given every 2 weeks for up to 12 cycles. In case of stable disease (SD) or partial response (PR), IFNα was continued until disease progression (PD). All pts were chemotherapy naive; one pt had prior treatment with $^{131}$I-metaiodobenzylguanidine.

***Results***: Patients received a median of 5 courses (range 1-12). Two pts (one carcinoid, one islet cell) achieved a PR (13%; 95% confidence interval 2-40%), of 3 and 4 months duration. Five pts (all carcinoid) achieved SD (33%) with symptomatic response, for a median of 8 months (range, 4-22 months). Both pts who achieved PR and 3/5 with SD had a > 50% marker reduction (5-HIAA, VIP). Four pts had PD. Another 4 were not assessable for response due to early toxicity necessitating cessation of treatment. Two had grade III-IV diarrhoea and 2 grade IV neutropenia requiring i.v. antibiotics. Four patients required a 50% dose reduction of the IFNα for fatigue.

***Conclusions***: These results suggest no clear advantage for the combination of 5FU and IFNα over the individual agents alone; the combination *may* also result in increased toxicity and reduced tolerance.

### PO43

PACLITAXEL, VINBLASTINE, CISPLATIN (PVC) IN PATIENTS WITH ADVANCED TRANSITIONAL CELL CANCER (TCC)

B McLaren[1], CJ Gallagher[1], M Mason[2], G Melesi[1], RTD Oliver[1], Depts of Medical Oncology, St Bartholomew's Hospital[1], London and Clinical Oncology, Velindre Hospital[2], Cardiff, United Kingdom.

Cisplatin based combination chemotherapy is standard treatment for pts with TCC however paclitaxel has been shown to have significant single agent activity (ASCO;1994 704). This study was initiated to pilot these two active agents combined with vinblastine.Eligibility criteria were advanced TCC, Karnofsky score >60, no prior systemic chemotherapy, adequate haematologic/ renal function. Treatment was paclitaxel 175mg/m$^2$ over 3 hr, cisplatin 70mg/m$^2$ day 1, vinblastine 3mg/m$^2$ days 1 and 8 repeated every 21 days with premed, dexamethasone, chlorpheniramine, cimetidine. Vinblastine was omitted on day 8 if total neutrophil count < 1.0. Fifteen pts were treated (13M, 2F).Median age was 66(54-75). Six had had radical tumour resection, eight transurethral tumour resection and one was inoperable. Five had received bladder radiotherapy Sites of disease; lymph nodes(10), bladder(6), lung(5), liver(3), pelvic mass(2), bone(2), ureter(1) and prostate(1). A median of 5 cycles was given (1-6) with 2 (13%) pts achieving CR and 5 (33%) PR. Overall response rate 47% (95% CI 22-72%). 3 pts had SD, 4 PD and 1 early death due to bowel obstruction. All responses were in locally recurrent tumour and/or lymph nodes. Grade III/IV neutropenia was observed in 14/67 cycles and 7 episodes of neutropenic fever occurred. Other grade III toxicity; alopecia(10pts) diarrhoea(2), constipation consistent with bowel obstruction(2), peripheral neuropathy(1), pain secondary to myalgia(1) and nephrotoxicity(1). Six patients had grade II paraesthesiae.Median time to progression was 6 mths. PVC is active in advanced TCC, with manageable toxicity.A future study is planned of MVAC vs MVPC substituting paclitaxel for doxorubicin.

### PO44

BONE SCINTIGRAPHY IN PATIENTS WITH CARCINOMA OF THE BLADDER REFERRED FOR RADICAL RADIOTHERAPY,

A.N.J Tutt, G.Jay, D.P.Dearnaley, Urological Oncology Unit, Royal Marsden Hospital, Sutton, SM2 5PT.

Purpose: To assess the value of a selective policy of use of scintigraphic bone scan (SBS) in patients referred for radical radiotherapy for transitional cell carcinoma (TCC) of the bladder.

Method: A retrospective review of 323 patients referred to our unit between January 1991 and August 1994 revealed 101 on whom SBSs had been performed based on the prospectively defined criteria of either, symptoms compatible with bone metastases or elevation of serum alkaline phosphatase (AP). Following independent review of the SBSs by a nuclear medicine radiologist, a possible correlation between positive SBS and raised AP was sought using a 2x2 table and chi squared analysis.

Results: Serum AP was elevated in 26% of those patients who had SBS. An SBS diagnosis of bone metastasis was made in 26% of patients on whom SBS was performed. An elevated serum AP was associated with a positive SBS in 50% . A normal serum AP was associated with an abnormal SBS in only 16% ($\chi2=16.4, p<0.0001$). Bone metastases were found predominantly distributed in the axial skeleton with no predilection for the lumbar spine or pelvis. Three patients with apparently solitary pelvic bone metasasis, who were treated radically, relapsed with multiple bone metastases within four months of completing treatment.

Conclusion: The selective use of SBSs, in patients being considered for radical treatment for muscle invasive TCC bladder, diagnosed bone metastasis in a quarter of those tested. The detection of an apparently solitary deposit should not influence the decision to treat such patients palliatively.

### PO45

A PHASE II STUDY OF SUBCUTANEOUS (SC) INTERLEUKIN-2 (IL-2), ALPHA INTERFERON (IFN) AND PROLONGED VENOUS INFUSIONAL (PVI) 5-FU IN PATIENTS WITH METASTATIC RENAL CELL CANCER.

MM Vaughan, SRD Johnston, JS Moore, ME Gore. Royal Marsden Hospital, Fulham Road, London SW3 6JJ

Biochemotherapy regimens which combine IFN, IL-2 and bolus 5-FU have been reported to have response rates of >40% in patients with renal cell cancer (Aztipodean et al. 1993). PVI 5FU has been shown to have advantages over bolus administration. In this study we assessed the efficacy and toxicity of PVI 5-FU with SC IL-2 and IFN. Twenty one patients with metastatic renal cell cancer were treated with SC IL-2 (10 MIU/m$^2$ bd days 3, 4 and 5 of weeks 1 and 4; and 5 MIU/m$^2$ days 1, 3 and 5 of weeks 2 and 3), SC IFN (6MIU/m$^2$ day 1 of weeks 1 and 4, and days 1, 3 and 5 of weeks 2 and 3; and 9 MIU/m$^2$ days 1, 3 and 5 of weeks 5-8) and PVI 5-FU (200 mg/m$^2$/day weeks 5-9). Patients with an objective response (CR/PR) or disease stabilisation were given a further 9 week cycle provided that toxicity was acceptable. Eight of 21 patients had a partial response in measurable sites of disease (38%, 95% CI 18-62), and there were a further 2 patients in whom disease was stable for at least 4 months. Five patients received less than 3 weeks treatment; 2 withdrew because of toxicity and 3 because of early disease progression. The overall median time to disease progression was 16 weeks (range 1 to 46), but this has not yet been reached in responding patients. All patients experienced grade 2 constitutional symptoms, and 5 patients suffered grade III toxicity (rash, 2 pts; hypotension, 1 pt; renal failure, 1 pt; confusion, 1 pt), this was reversible in all patients. In conclusion, IL-2, IFN and PVI 5FU is a promising and active biochemotherapy regimen in metastatic renal cell cancer.

Reference:

Aztipodean J et al. Interleukin-2 in combination with interferon alpha and 5-Fluorouracil for metastatic renal cell cancer. Eur J Cancer 1993; 29A (Supp 5): S6-8.

### PO46

COMPARATIVE TOXICITY OF VP 16-213 IFOSFAMIDE AND CISPLATIN (VIP) PLUS HIGH DOSE VERSES TAXOL IFOSFAMIDE CISPLATIN (TIP) PLUS HIGH DOSE IN SEQUENTIAL PHASE 1/2 STUDIES.

CA O'Doherty, S Asterling, J Ong, CJ Gallagher, RTD Oliver. Medical Oncology, St Bartholomew's Hospital, London EC1A

Currently three courses of VIP plus a single high dose treatment with peripheral blood stem cell rescue is the experimental arm in a pan European randomised trial against 4 courses of VIP as first line salvage therapy for germ cell cancer patients failing BEP therapy. Prompted by reports that Taxol had activity in germ cell cancer patients we have set up a study investigating the effects of substituting Taxol 210mg/m2x1 for Bp 16-213 125mg/m$^2$ dl-3 in the induction protocol. This abstract reviews the comparative of our sequential phase 2 experience of these two regimens. A total of 22 patients have been induced with VIP and 17 with TIP. Six in each study did not procede to high dose, either because they were drug resistant, or had chemo responsive disease with residual mass that was sufficiently localised for surgery. Twenty seven patients proceeded to high dose procedure, there were two major episodes. One of these was renal failure on requiring dialysis after VIP and the second was bowel necrosis after TIP. Apart from this there were no treatment related mortality or morbidity. There were no significant differences in time to neutrophil or platelet recovery or time to discharge. 7 of 16(44%) of VIP+HD remain relapse free for more than 22 months, while 9 of 11 (82%) with TIP remain progression free after a median of 6 months. It is concluded that it is possible to substitute Taxol 210mg/m$^2$ for Etoposide in the VIP plus high dose regiment without producing any major increase in toxicity and that responses at this stage seem to be at least equivalent to those achievable with VIP and that further study of this approach is justified.

### PO47

**MALIGNANT SUPRATENTORIAL GLIOMAS IN CHILDHOOD: THE GREAT ORMOND STREET EXPERIENCE.**

N. Shah[1], K. Phipps[2], A. Michalski[1,2], W. Harkness[2], R. Hayward[2] and M. Gaze[1,2]. [1]The Middlesex Hospital and [2]The Great Ormond Street Hospital for Children (GOSH), London.

***Introduction*** High grade supratentorial gliomas in children account for 7-11% of primary brain neoplasms. Unlike their adult counterparts, longer survival is noted following standard treatment. This retrospective study analyses survival in relation to patient and tumour related factors, and treatment techniques in children referred to the GOSH from 1980-1995.

***Method*** 52 patients with histologically proven high grade supratentorial gliomas were reviewed. The median age of presentation was 7.25 years (range 2 months-15.75 years) with a male to female ratio of 30:22. The ratio of Grade 3 to Grade 4 tumours was 22:30. The table summarises the various treatments employed.

| Primary treatment | Surgery | RT | CT + RT | CT |
|---|---|---|---|---|
| Open Biopsy | 4 | 3 | 0 | 0 |
| Steretactic Biopsy | 6 | 5 | 0 | 0 |
| Incomplete resection | 36 | 27 | 3 | 2 |
| Gross resection | 6 | 3 | 1 | 1 |

RT= Radiotherapy, CT= Chemotherapy,

***Results*** Overall median survival from the time of histological diagnosis was 29 months (range 0-14.4 years).The proportion of patients alive at 2 and 5 years following treatment were 52% (95% CI 37-67%)and 37% (95% CI 20-54%) respectively. Improved survival was not noted with the extent of resection (p=0.39), radiotherapy (p=0.48), grade of tumour (p=0.86) or age of the patient (p=0.27).

***Conclusions*** In our series, the median survival is comparable to the published literature assessing childhood gliomas. In contrast, we have not observed a survival benefit with macroscopic resection of the tumour or post operative radiotherapy.

### PO48 POSTERIOR FOSSA BOOST IN MEDULLOBLASTOMA - A COMPARISON OF STANDARD FIELD AND THOSE PLANNED FROM THE PRE-OPERATIVE MRI SCAN

I D Pedley, R E Taylor, G E Gerrard - Yorkshire Regional Centre for Cancer Treatment, Cookridge Hospital, Leeds, West Yorkshire

**Introduction:** Craniospinal radiotherapy followed by a posterior fossa boost is the routine management for medulloblastoma. The standard posterior fossa field in the current SIOP PNET 3 Study is delineated by the posterior clinoids anteriorly, the inner table of the skull posteriorly, the $C_2/C_3$ interspace inferiorly and superiorly 1cm above the midpoint between the foramen magnum and the vertex. The object of this study was to investigate whether adopting ICRU 50 guidelines with the pre-op MRI images would alter the posterior fossa field size.

**Methods:** For 17 patients with medulloblastoma the gross tumour volume (GTV) was marked on the orthogonal film as defined by the $T_2$ weighted image. Using a 2cm margin and applying the ICRU 50 guidelines the field size was obtained and compared to the standard.

**Results:** In 13 patients the field sizes were identical. For 3 patients the MRI derived fields were smaller than the standard and in all cases this was due to the inferior margin. The mean difference was 8mm (range 6 to 10mm). In the one other patient the MRI derived field was larger by 9mm due to the inferior margin.

**Conclusion:** In the majority of cases the posterior fossa field is delineated by the standard field. However it is recommended that in all cases the pre-operative MRI scans be studied to ensure adequate coverage of the GTV and also to exclude the possibility of using smaller fields.

### PO49 A COMPARISON OF TREATMENT VOLUMES PLANNED USING CT AND MRI IMAGING IN THE TREATMENT OF CEREBRAL GLIOMAS.

G.E. Gerrard, R.E. Taylor, M.P. Collinson, I.D. Pedley, J. Povall. Dept. Of Clinical Oncology, Cookridge Hospital, Leeds, West Yorkshire.

The object of this study was to compare the treatment volumes planned using MRI imaging with those obtained using CT scans.

Materials and methods: The study involved twenty patients with cerebral gliomas [ twelve low grade and eight high grade gliomas] who received radical radiotherapy at either Cookridge hospital, Leeds or Weston Park hospital, Sheffield between June 1995 and December 1996. The pre-operative gross tumour volume [GTV] was marked on the orthogonal antero-posterior and lateral radiographs, using initially the CT images then the MRI information. The contrast enhancing edge on CT was used to define the GTV [or the hypodense area if non-enhancing]. With MRI images the enhancing edge with gadolinium was used to define the GTV [or the T2-weighted image if there was no enhancement]. The target volume was then marked and a treatment plan produced. Field sizes were taken from the mid-plane of the plan and the irradiated volume calculated [as defined by 50% of the intersection dose].

Results: In twelve patients the CT and MRI volumes were identical. In six patients the MRI volume was larger than the CT volume, with an average size difference of 105 cm3 [ range 68 to 145 cm3]. However the field sizes were only 0.5 to 1.0 cm larger in one or two dimensions. These patients had little or no enhancement with gadolinium. Smaller volumes with MRI imaging were found with two patients with low grade gliomas which were hypodense on CT scanning, and did not enhance following contrast with either CT or MRI. In these two cases the MRI volumes were 241cm3 and 270 cm3 smaller than the CT volumes, corresponding to a 0.5 to 2 cm smaller field sizes in two dimensions. For all twenty patients the MRI derived mean irradiated volume was 1412 cm3 [ range 319 to 2561cm, 95% confidence interval +/-303 cm3], and the CT derived mean irradiated volume was 1405 cm3 [ range 319 to 2760 cm3, 95% confidence interval of +/-298 cm3].

Conclusions: Since in the majority of patients [60%] the two volumes were identical there seems no need to routinely use different margins with MRI and CT imaging. In the cases [30%] in which the MRI volumes were greater, the resulting field sizes were only 0.5-1.0 cm in 1-2 dimensions. MRI was found to be preferable in low grade tumours which showed no enhancement on CT and resulted in smaller irradiated volumes.

### PO50 BENIGN MENINGIOMA OF THE SKULL BASE.

C Nutting, L Brazil, A Sibtain, J Steele, H Alheit, D Traish, S Ashley, M Brada. Neuro-oncology Unit & Academic Unit of Radiotherapy & Oncology, Institute of Cancer Research & Royal Marsden NHS Trust, Sutton SM2 5PT, UK.

**Purpose:** To assess the long-term results of conservative surgery and conventional external beam radiotherapy (RT) in the management of skull base meningioma, particularly as a baseline to help in the evaluation of new treatment strategies.

**Patients and methods:** A retrospective review of 82 patients treated at the Royal Marsden Hospital for benign meningioma of skull base between 1962-1992.

**Results:** 82 patients aged 13-74 (median 50) years with benign skull base meningioma were treated with conservative surgery and radiotherapy. 62 patients received RT after initial diagnosis and 20 after tumour recurrence. 33 patients had sphenoid ridge, 26 suprasellar and 23 other skull base tumours. Initially 73 patients had subtotal excision or biopsy and 9 complete excision. The usual radiotherapy was 50-55 Gy in 33 fractions (median 50.8 in 33 F). 10 year progression free survival (PFS) was 83% with site of disease, the only independent prognostic factor on multivariate analysis. Tumours of the sphenoid ridge had significantly worse disease control than other sites (69% vs 90-100% 10 years PFS). 10 year survival was 71% with PS (+age) the only significant prognostic factor.

**Conclusion:** Although the efficacy of RT in the treatment of incompletely excised benign meningioma remains unproven, the majority of patients achieve excellent long-term tumour control, particularly young patients with good PS, and tumours outside the sphenoid. The results can be used as a baseline for the evaluation of new treatment strategies. We are now evaluating stereotactic RT in this setting and have treated 17 patients in the last year.

### PO51 THE VEDEX REGIMEN: AN EFFECTIVE AND WELL TOLERATED PALLIATIVE TREATMENT FOR RELAPSED NON-HODGKIN'S LYMPHOMA (NHL).

L El-Helw, PC Lorigan, RE Coleman and BW Hancock. YCRC Department of Clinical Oncology, Weston Park Hospital, Sheffield, S10 2SJ, UK.

We have evaluated the efficacy and toxicity of a novel weekly intravenous palliative chemotherapy regimen with vincristine 1mg, epirubicin 30mg/m$^2$ and dexamethasone 20mg (VEDex) in relapsed NHL. This was a retrospective study of 49 consecutive patients treated at Weston Park Hospital. The median age was 68 years with a range of 34 to 88 years. 17 patients (34.7%) had low grade disease resistant to conventional alkylating therapy and 3 patients (6.1%) had transformed (initially low grade) NHL. 29 (59.2%) had relapsed high grade NHL; of these 22 had poor performance status which precluded high dose chemotherapy and 7 were heavily pretreated. Responding patients received up to a maximum of 8 cycles of treatment but this could be repeated at a later stage if required. The overall response rate was 67.3%, 10 patients (20.4%) achieved complete and 23 (46.9%) partial response. A further 16 patients (32.7%) had stable disease. 23 patients (46.9%) reported complete resolution of symptoms and 15 (30.6%) had partial resolution of symptoms. Grade III neutropenia was seen in 7 patients (14.3%) and grade IV in 1 (2%). Other relevant toxicities included nausea and vomiting grade III (4.1%) and alopecia grade III (2%). Peripheral neuropathy of greater than grade I was not reported. The median survival from start of treatment was 6 months. No patients died of treatment related toxicity. VEDex is an effective and well tolerated palliative treatment for patients with relapsed NHL who are elderly or have a poor performance status and/or who are heavily pre treated.

### PO52 PRIMARY INTRACEREBRAL LYMPHOMA : A CLINICAL STUDY OF 22 PATIENTS.

R.E.Hough, M.H. Robinson, P. Lorigan, B.W.Hancock. YCRC Department of Clinical Oncology, Weston Park Hospital, Sheffield.

Primary intracerebral lymphoma is an uncommon presenting site for non-Hodgkin's lymphoma. The authors review 22 histopathologically confirmed, consecutive cases presenting over a 15 year period between January 1982 and January 1997.

The cohort included 16 males and 6 females with a mean age at diagnosis of 55.0 years (range 27-75 years). 2 patients were taking immunosuppressive therapy following renal transplantation. Presenting symptoms included personality change and confusion (50%), headache (40%), limb weakness (30%), seizures (20%) and visual disturbance (20%). The mean time from onset of these symptoms to diagnostic biopsy was 8.5 weeks. Subtotal resection was performed in 5 patients. Radical whole brain irradiation was given to 20 patients; 2 received additional radiation to the tumour site. 2 patients were too unwell to receive treatment and subsequently died. 3 patients also had chemotherapy. Clinical remission was achieved in 14 patients. Of these, 7 relapsed after a median time of 10 months - 4 received palliative chemotherapy and 1 of these attained a further remission. 8 patients (36% total cohort) are still alive and in remission after a median period of 2 years and 8 months (range 6 months to 10 years and 10 months). Cause of death was intracerebral lymphoma in 11 of the 14 patients who died, pulmonary embolus in 2 and bronchopneumonia in 1. Median survival was 5 months in this group.

Primary intracerebral lymphoma is a rare tumour associated with poor prognosis. However, durable remission can be achieved with radical treatment (primarily radiotherapy) at presentation.

### PO53 PRIMARY NON HODGKINS LYMPHOMA OF BONE - TREATMENT AND OUTCOME

S Sothi and D Spooner , Royal Orthopaedic Hospital and Queen Elizabeth Hospital, Birmingham, U.K.

Primary non Hodgkins lymphoma of bone is uncommon. Between 1985 and 1996, 32 patients presenting with primary bone lymphoma were treated at our centre. Diagnosis was confirmed histologically in all patients. 29 patients had high grade lymphoma, 3 low grade. 31 had B cell tumours , one T cell. Mean age of patients was 48 years (range 5 to 89 years). 19 patients (56 %) presented with disease localised to a solitary bone (Stage IE), 2 patients had solitary bone disease with regional nodes (Stage IIE) and 10 patients had Stage IV disease. One patient had recurrent disease. Of the Stage IV disease, 4 had multifocal bone disease, 2 had distant nodal disease and 4 had other extranodal sites of involvement. 18 patients had long bone tumours, 14 had axial tumours.

25 patients were treated with chemotherapy (standard CHOP regime) followed by local radiotherapy . 3 patients had surgery as their local treatment (endoprosthetic replacements, fibulectomy) and chemotherapy. 2 patients had chemotherapy alone ( both children ). 2 patients had surgery (spinal decompression), chemotherapy and radiotherapy.

29 cases completed the prescribed treatment and are evaluable for response. All Stage IE patients who completed chemotherapy and radiotherapy achieved complete remission with no evidence of local recurrence (mean follow up of 46 months , range 6 to 133 months). Of the Stage I patients, 1 out of 19 developed systemic disease (low grade). Of the Stage IV patients, 2 out of 10 died of progressive lymphoma. Five year survival using the Kaplan Meier survival analysis was 84 % for Stage IE patients and 42% for Stage IV patients.

The addition of chemotherapy to local treatment for primary lymphoma of bone has improved outcome.

### PO54 IVE SALVAGE CHEMOTHERAPY AND MOBILISATION OF PERIPHERAL BLOOD STEM CELLS (PBSC) FOR RELAPSED HODGKIN'S AND NON-HODGKIN'S LYMPHOMA.

L.K. Dawson(1), J. Craig(2), D.A.Cameron (1), F. Forrest (1), and R.C. F. Leonard(1). (1) Dept. of Clinical Oncology, Western General Hospital, Edinburgh, EH4 2XU. (2) Blood Transfusion Service, Royal Infirmary, Edinburgh.

Salvage therapy in lymphoma comprises a variety of regimens with the intention of not only achieving further disease control but potentially to provide an opportunity to harvest stem cells for subsequent treatment intensification to consolidate the response obtained. The IVE regime ( Ifosfamide 3g/m2 days 1-3; Epirubicin 50mg/m2 day 1 and Etoposide 200mg/2 days 1-3 ) in conjunction with G-CSF was given for a maximum of 3 cycles in 11 patients aged 20-70 years.

Of the 11 patients treated from 1994-1997, 2 had nodular sclerosing Hodgkin's disease (HD); 9 had intermediate and high grade non-Hodgkin's lymphoma (NHL). All had had a minimum of one previous chemotherapy regimen and at best had achieved a partial response to therapy.

The treatment was tolerated reasonably well by all ; only 1 patient had problems with Ifosfamide induced encephalopathy ; 8 patients required inpatient treatment for neutropenic sepsis with 3 patients requiring either discontinuation or modification of therapy ; 4 patients did not require blood product support during treatment.

Successful mobilisation of PBSC was obtained in all 9 patients considered for further high dose chemotherapy. 5 patients have been treated and successfully engrafted, 1 is patient currently starting treatment following re-induction after aggressive relapse and 1 patient is under assessment. The other 2 patients were not felt to be suitable for consolidation high dose treatment.

### PO55 ACQUIRED IMMUNODEFICIENCY SYNDROME (AIDS) - RELATED PRIMARY CEREBRAL LYMPHOMA : RESPONSE TO IRRADIATION.

V. S. Khoo[1], K. H. Liew[2] and M. Sexton[2]. [1]Academic Unit of Radiotherapy, Royal Marsden NHS Trust, Sutton, Surrey SM2 5PT, [2]Dept. of Radiotherapy, Peter MacCallum Cancer Institute, East Melbourne, Australia 3002.

Aim and methods: Fourteen cases of AIDS-related primary cerebral lymphoma (APCL) were retrospectively reviewed between 1986 and 1994 to characterise the natural history and response of these patients to radiotherapy (RT). Results: The median age was 38 years (range 24-65). The median interval between sero-positive diagnosis of the human immunodeficiency virus (HIV) and APCL was 28 months (range 5-113). The median duration of symptoms prior to presentation was 2 weeks (range 0.2-12). At presentation, 12/14 patients were ECOG performance status 2 or 3. The symptoms and signs were non specific. There was no consistent specific pattern of brain imaging in terms of size, number, location or pattern of contrast enhancement of the cerebral lesions. Nine patients were selected for RT based on their performance status and received varying fractionation schedules (2000-5000cGy). The response rate was 89% (8/9) with either improvement or stabilisation in neurological symptoms or performance status. Unfortunately the response duration was short with a median survival time (MST) post-RT of 5 weeks (range 0.6-37). The MST from presentation at our institution for treated and untreated patients were 8.5 and 2 weeks respectively (range 0.9-43) (p=0.004). Conclusion: The optimum management for APCL remains undefined. Although patient selection introduced bias and influenced the outcome, there appears to be a modest improvement in MST for treated patients. The survival results using RT alone for A-PCL remains poor but RT may provide substantial palliation. For some selected patients, a prolonged response is possible.

### PO56

HIGH-DOSE CHEMOTHERAPY AND AUTOLOGOUS STEM CELL TRANSPLANTATION IN 3 PATIENTS WITH LYMPHOMATOUS POLYPOSIS R. A. Popescu and D. Cunningham *Department of Medical Oncology, The Royal Marsden NHS Trust, Sutton, Surrey*

Lymphomatous polyposis (LP) is a rare, distinctive type of primary B-cell gastrointestinal (GI) lymphoma, characterised by multiple polyps affecting various parts of the GI tract, and thought to be similar in genotype, phenotype and clinical behaviour to nodal mantle cell lymphoma (MCL). The classical histological appearance is of small cleaved centrocytes arranged in a nodular pattern of variable degree, which are CD20+, CD5+, CD23-, CD10-, CD3-. Despite being classified as a low-grade lymphoma, LP has a poor response to chemotherapy (including anthracyclin-containing regimens), with short remissions and median overall survival. Ongoing chemotherapy trials for MCL attempt to maximise response rates and duration by chemotherapy dose intensification. We report our experience with high-dose treatment in 3 male patients with LP. 2 patients had ileum or colon resections at diagnosis. All patients achieved remission (2CR, 1PR) following conventional chemotherapy (2 CHOP, 1 Pacebom), lasting 4-16 months. They relapsed systemically and received salvage chemotherapy (EPIC, DHAP, CHOP). Two patients re-attained a PR, and one a near CR. High dose chemotherapy and stem cell rescue was given, following which all 3 went into CR. One patient relapsed 7 months later in the spleen, which was involved, as was his bone marrow, at first relapse. He responded poorly to 4 further lines of treatment, and died 23 months following transplant. The two other patients remain well and in CR 6 and 54 months following transplant. High-dose chemotherapy and autologous transplantation for LP is feasible and may induce long remissions in a disease conventionally thought to be incurable, in analogy to follicular lymphoma where strong evidence for this has accumulated. Further studies are needed to determine efficacy and role of high-dose therapy and autologous support in the management of lymphomatous polyposis.

### PO57

**VARIATION IN SPINAL CORD DOSE IN PATIENTS RECEIVING PALLIATIVE RADIOTHERAPY FOR LUNG CANCER** **D.Bottomley, A Morgan, D Sebag-Montefiore, E McVey, H Probst, D Dodwell. Yorkshire Centre for Cancer Treatment,Cookridge Hospital, Leeds.**

**Introduction** The publication of the MRC LU08[1] trial has led to the widespread use of a fractionation of 17 Gy in 2 fractions one week apart for patients with inoperable lung cancer. The radiobiological analysis of the three reported cases of radiation myelitis in the MRC trials[2] was based on the assumption that the average dose to the cord might be 5% greater than midplane and recommends that if the thoracic spinal cord dose exceeds a linear quadratic equivalent dose ($LQED_2$) of 48 Gy that methods to reduce the cord dose should be considered. A pilot study was therefore designed to assess the variations that exist in the maximum spinal cord doses depending on patient separation and machine energy.
**Methods** Fifteen patients with inoperable lung cancer who received 17 Gy in 2 fractions one week apart using parallel opposed fields were studied. In addition to the conventional anteroposterior simulator film, a lateral simulator film was also taken to determine patient separations and the depth of the spinal cord. The central axis, superior and inferior separations were recorded by a radiographer. The maximum spinal cord dose was calculated without lung correction for a $^{60}$Co unit and 6MV and 8MV linear accelerators. The maximum spinal cord doses were used to calculate the $LQED_2$ using an alpha/beta ratio of 2Gy.[2]
**Results** The mean patient seperations were 19cm (range 17-23.5cm) at the central axis,18.5cm at the superior field border (range 14-21.5cm) and 21.5cm at the inferior field border (range 18.5-25.5cm). The mean maximum cord doses were 5.1% (range 2.2%-7.5%), 7.3% (range 4.1%-11.6%) and 10.3% (range 3.1%-16.1%) higher than the prescibed midplane dose for 8MV, 6MV and $^{60}$Cobalt respectively. An $LQED_2$ of greater than 48Gy was found in 14 (83%) of patients using $^{60}$Co, 15 (100%) patients using 6MV and 10 (67%) of patients using 8MVrespectively.
**Conclusions** This small study demonstrates greater variations in the maximum cord dose than expected. Maximum spinal cord doses are dependent on separation and machine energy and clinicians need to take account of these factors when deciding whether steps should be taken to reduce the maximum dose to the spinal cord.

[1] Medical Research Council Working Party. Br J Cancer 1991;63:265-270
[2] McBeth et al Clinical Oncology 1996;8:176-181

### PO58

WITHDRAWN

### PO59

**A PHASE II OF CISPLATIN (CDDP), IFOSFAMIDE (IFM) AND INCREASING DOSAGE OF NAVELBINE (NVB) IN UNRESECTABLE NON-SMALL CELL LUNG CANCER (NSCLC)**

P.J. Souquet[1], P.Fournel[2] - [1]Service de Pneumologie CH, Pierre Benite Lyon-France, [2] Service de Pneumologie CH St Etienne-France.

CDDP, IFM and NVB are the most active drugs in NSCLC, as single agent. NVB has already shown an increased survival rate in randomised trials. In particular, one of such trial showed the combination of NVB and cisplatin to result in statistically superior survival compared with standard therapy (JCO 1994), This was recently confirmed by the SWOG study comparing NVB+CDDP vs CDDP (ASCO 1966). Preliminary results with NVB+CDDP+IFM are very encouraging. Based on this data, since 12/92 to 02/94 a clinical trial was performed in patients(pts) with unresectable stage III (IIIA N2, IIIB) NSCLC treated with 3 different schedules : __Group A__ : CDDP (75mg/m² D1)+IFM (3g/m² D1)+NVB (25mg/m² on D1) ; __Group B__ : CDDP (75mg/m² D1)+IFM (3g/m² D1)+NVB (25mg/m² on D1 & 8) ; __Group C__ : CDDP (75mg/m² D1)+IFM (3g/m² D1)+NVB (25mg/m² on days 1 & 15 and 12.5mg/m² on day 8) every 21 days. The purpose was to assess the response rate (RR), survival and tolerance. A first assessment, according to WHO criteria was done after 3 cycles. After this, stage III patients received standard radiotherapy (60Gy over 6 weeks) and responding stage IV pts received 3 cycles more.
85 pts were included (A: 35 pts; B: 28 pts; C24 pts): median age 59 y. (range 36-73) males 74 pts, stage III 37 pts; stage IV 48 pts. 32 pts suffered from adencarcinoma, 37 from squamous cell and 16 from undifferentiated cell.
Dose intensity (according to Hryniuk method) was the same for the 3 groups concerning CDDP and IFM. For NVB dose density was 8.1mg/m²/w, 14.7mg/m²/w and 16.9mg/m²/w for A,B,C groups respectively. Haematological toxicity was predominantly observed, mainly in the group B.
5 pts achieved complete response (CR), 36 pts partial response (PR) after 3 cycles but 14 CR and 17 PR at the end of treatment. The overall median survival( MS) 40 ws. The RR observed by group was 31.5% (30 ws MS), 44.5% (40 ws MS), 66.6% ((55 ws MS) for group A,B and C respectevely.
This study confirms that increased dose intensity with. NVB is feasible and improves response rate and survival without haematological toxicity raise.

### PO60 NEOADJUVANT CHEMOTHERAPY WITH NAVELBINE (NVB) PLUS CISPLATIN (C) IN STAGE $III_B$ IN NON-SMALL CELL LUNG CANCER (NSCLC): A PHASE II TRIAL

Cigolari S.[1], Curcio C.[2], Massimo M.[3], Sessa R.[3], Vasta M.[2], Maiorino A.[3]
[1]Universita Federico II, [2]Hospital Ascalesi, [3]Hospital Monaldi (Napoli, Italy).

NVB+C has already proven to be one of the most effective regimen in NSCLC in terms of survival for patients (pts) with unresectable disease (Le Chevalier, JCO, 1994). Based on these results we planned to treat pts with stage IIIB NSCLC ≤ 75 years(y) old, in order to determine response rate (RR) and operability of locally advanced disease.

***Patient's characteristics:*** between Apr.1996 and Nov.1996, 30 pts were accrued: 28 of them were evaluable (1 too early, 1 lost to follw-up); median PS 1; median age 61 y (38 - 75).

***Treatment:*** NVB 30 mg/m² on D1 and D8 and C 120 mg/m² on D1 in a 21-day schedule for 3 cycles before restaging. G-CSF was permitted in case of severe neutropenia.

***Results:*** A total of 81 cycles have been administered with mainly myelotoxicity: anemia grade(G) 3: 13%, leucopenia G3: 16%; no thrombocytopenia. Clinical toxicity was mainly nausea and vomiting G3 in 66%.

The efficacy after 3 cycles was: 16/28 (57%) partial response, 8 of them were resected, all responders were confirmed by an independant external expert group; 6/28 no change and 6/28 progressive disease.

***Conclusions:*** This NVB+C as a neoadjuvant schema showed a high RR (57%), resulted in patients being able to proceed to resection and will be tried in a Phase III clinical study in order to study its possible effects on survival.

### PO61 CHEMOTHERAPY WITH VINORELBINE (NAVELBINE : NVB) + CARBOPLATIN IN ADVANCED NON-SMALL CELL LUNG CANCER (NSCLC)

B. Parente[1], F. Barata[2], J. Moura e Sa[1], R. Pato[2], L. Horta[1], J. Seada[1] - [1]Centro Hospitalar Vila Nova de Gaia, [2]Centro Hosp. de Coimbra - Portugal

Between Jan. 1995 and May 1996, 75 patients (pts) (56 and 19 from Vila Nova de Gaia Hospital and Coimbra Hospital respectively) with advanced NSCLC were treated with NVB (30 mg/m²) on days 1 and 8 and Carboplatin (300 mg/m²) on day 1, every 3 weeks (w). Pts were evaluated after 3 cycles and the treatment was continued for a further 3 cycles in case of tumor response.

**Patient's characteristics:** sex F/M: 11/64, median age 59 y (27-73), PS 1: 75%, PS 2: 25%; histology: epidermoid: 52%; adenocarcinoma: 44%; others: 4%. Stage IIIA, IIIB and IV: 1.4%, 69.3%, 29.3% respectively.

**Results:** The overall response rate was 45.3% with a median duration of 31 w; the median overall survival was 34 w; the median survival of responders was 43.5 w.

**Toxicity:** 332 cycles were administred and the limiting toxicity was mainly myelosupression: WHO Grade(G) 3 anemia: 4%; G3-4 neutropenia: 23%; G3-4 thrombocytopenia: 4%; acceptable non haematological toxicity: local reaction: 20% and no significative alopecia were observed.

**Conclusions:** This study confirms that this combination achieves a high response rate, good median survival with acceptable tolerance. This combination should be recommended for the treatment of pts with NSCLC on an outpatient basis.

### PO62 AN OVERVIEW OF 3 PHASE II TRIALS OF NAVELBINE (NVB), AND FRACTIONATED DOSES OF CISPLATIN (CDDP) IN THE MANAGEMENT OF ADVANCED NON-SMALL CELL LUNG CANCER (NSCLC)

M. Pawlicki[1], J. Rodrigues[2], D. Firat[3], F. Le Bras[4] & FM. Delgado[4]
1- Onc. Cent. Krakow-Poland; 2-ELAN-Brazil; 3-Ankara Univ. Turkey; 4- I.R.P.F., France.

**AIM**: The combination of NVB and CDDP has shown statistically superior survival compared with standard therapy (JCO 1994, ASCO 1996). 3 phase II studies were conducted to assess a new schedule of this combination which can be given on an out-patient basis: NVB 25 mg/m² (1 trial 30 mg/m²) on day 1 & 5 and CDDP 20 mg/m² daily over 5 days (D1-5) every 21 days, (maximum 6 cycles). **Results**: Between 7/94 and 2/96, 127 (pts) were included: median age 60 (34-75). 112 (88%) males; PS 0, 1 and 2, 16%, 55% and 27% respectively. squamous cell - 56% ,adenocarcinoma -36% and large cell -8%; 12% stage IIIA, 36% stage IIIB and 49% stage IV and 3% unknown (metastatic). 471 courses were administered (median 4, range 1-8). WHO grade (G) 3-4 neutropenia -12%; G3-4 infection episodes 1.4% of courses. G 3 nausea/vomiting: 18% (5.4% of courses). Only 4% of pts developed WHO grade 3 constipation and grade 3-4 peripheral neuropathy was observed in 9% of pts (2.4% G 4). G3 alopecia -12%. The **overall response rates** observed in Brazilian, Polish and Turkish studies are 46%, 47% (N 30 mg/m²) and 29% respectively; median TTP: 7.4 months and median survival is: 9.2 months **Conclusion**. These results confirm that NVB + CDDP in combination have constant and reproducible high antitumour activity in NSCLC. This new schedule seems well suited for use in the out patient management of NSCLC.

### PO63 THYROGLOBULIN ANTIBODIES IN DIFFERENTIATED THYROID CANCER.

P. Hjiyiannakis, J. Mundy, C. Harmer, Thyroid Unit, Royal Marsden NHS Trust, Downs Road, Sutton, Surrey SM2 5PT UK

**INTRODUCTION** A retrospective review of patients with differentiated thyroid cancer between 1984-1996, at The Royal Marsden Hospital, identified 40 patients with serum Thyroglobulin Antibodies (TgAb). These can interfere with the immunoradiometric assay (IRMA) for Thyroglobulin (Tg) used at this hospital, with a resultant underestimation of the Tg levels. Median follow up from diagnosis was 26 months (range 3-401 months); median age at diagnosis was 50 years.

**RESULTS** Patients were assigned to one of two groups: group 1 with TgAb>1/100 (n=28) and group 2 with TgAb<1/100 (n=12). Fourteen patients recurred: 12 in group 1 and 2 in group 2. Sites of recurrence were: neck (n=10), lung (n=5), bone (n=4), brain (n=2). None of the patients in group 1 showed an elevated Tg with recurrence. One patient in group 2, in whom the TgAb did not interfere with Tg assay, showed an appropriate Tg response with recurrence. Eight patients in group 1 had TgAb development apparently in response to tumour recurrence. Seven of these patients have died, with a median overall survival of 26 months from diagnosis. TgAb presence did not influence overall survival of the whole group or overall survival of patients with papillary carcinoma (n=34).

**CONCLUSION** We recommend that laboratories should routinely report the presence of TgAb, with a caution indicating the direction of possible error (which depends on the assay used). Development of TgAb can indicate tumour recurrence, which carries a poor prognosis. In TgAb positive patients serial monitoring may offer additional, clinically useful, information.